# A protocol for a critical realist synthesis of school mindfulness interventions designed to promote pupils’ mental wellbeing

**DOI:** 10.3389/fpubh.2023.1309649

**Published:** 2024-01-09

**Authors:** Pamela Abbott, Graeme Nixon, Isabel Stanley, Lucia D’Ambruoso

**Affiliations:** ^1^Centre for Global Development, University of Aberdeen, Aberdeen, United Kingdom; ^2^School of Education, University of Aberdeen, Aberdeen, United Kingdom; ^3^Aberdeen Centre for Health Data Science, University of Aberdeen, Aberdeen, United Kingdom

**Keywords:** mindfulness, schools, mental wellbeing, children and adolescents, pupils, critical realism, complexity theory

## Abstract

**Introduction:**

The review described in this protocol will be the first critical realist review of the literature reporting on the impact of school-based mindfulness interventions on the mental wellbeing of pupils. Mindfulness interventions are increasingly being introduced into schools to promote children’s (and teachers’) wellbeing. Findings from impact evaluations, including systematic reviews and metanalysis, suggest that school-based mindfulness interventions promote pupils’ wellbeing. However, there is a need for further evidence on the underlying causal mechanisms and contexts that explain program outcomes, to provide insight into how mindfulness programs can be successfully implemented in other contexts.

**Methods and analysis:**

A critical realist review methodology will be used to provide a causal interdisciplinary understanding of how school-based mindfulness interventions promote the mental wellbeing of pupils. This will be done through a systematic literature review and extrapolating context, agency, intervention, mechanisms, and outcome configurations. This will enable an understanding of how, in certain contexts, pupils can use the resources offered by a mindfulness intervention knowingly or unknowingly to trigger mechanisms that promote their mental wellbeing and what mechanisms in the context support, restrict or prevent change. We will then use retrodiction and retroduction to develop the most plausible interdisciplinary middle-range theory to explain the findings.

**Discussion:**

The review findings will inform a critical realist evaluation of a mindfulness intervention in schools. The findings from the review will also enable us to inform policymakers and other stakeholders about what conditions need to be in place for mindfulness interventions to promote pupils’ mental wellbeing. We will publish the findings from the review in academic and professional publications, policy briefs, workshops, conferences, and social media.

**PROSPERO registration number:** CRD42023410484.

## Introduction

This protocol sets out how we will carry out a critical realist synthesis review of the literature on the impact of universal school-based mindfulness interventions (SBMIs) integrated into the school curriculum to promote pupils’ mental wellbeing (1). Our approach is grounded in a commitment to social justice to the pursuit of practical knowledge that will promote children’s and young people’s wellbeing and their well-becoming, enabling them to flourish (2–5). The aim is to build an explanatory theory of how universal SBMIs have the impact they do, enabling us to build transferable integrative theories that can inform the design, implementation and uptake of universal SBMIs (6–9).

Schools are an appropriate place to promote children’s and adolescents’ health and wellbeing because of the time they spend in school, allowing teachers to influence their health positively, and because of the pivotal role school plays in their lives (10–14). Universal interventions target all pupils in a defined population (e.g., a class, an age group or all the pupils in a school), recognize that universal provisions are non-stigmatizing, avoid the ‘prevention paradox’ (15), are relatively low-cost per pupil, attempt to reduce a number of risk factors and promote a broad range of protective factors, and may have effects on promotion, prevention and treatment (16, 17). They may also prevent mental, emotional, or behavioral disorders that develop in later life but are not manifest among school pupils (17).

In response to global concerns about the mental wellbeing of children and young people (18–20), SBMIs are increasingly being used variously to promote pupil’s wellbeing, executive functioning and resilience, promote their mental health, prosocial behavior and healthy relationships, and improve academic performance and the classroom and school climate (21). This increase in delivery has been accompanied by a significant increase in the publication of the findings from impact evaluations of SBMIs since 2000, with the numbers accelerating since 2010 (22, 23). The evidence for the effectiveness of SBMIs, including universal ones, as evidenced from the findings from impact evaluations (including high-quality trials, systematic reviews and metanalysis), is promising, suggesting that they are safe and effective for promoting pupils’ mental wellbeing (Supplementary material 1). Research, albeit limited, also indicates that SBMIs are generally acceptable to pupils and that they have experienced benefits from using mindfulness techniques (24–31).

While systematic reviews and meta-analyses have indicated promise regarding impacts, but outcomes have varied. Not all interventions have been shown to work (32–35), and not all mental wellbeing outcomes are addressed through mindfulness (21, 36). There is no agreed definition of what a school-based mindfulness intervention is (37, 38); they can be delivered by external facilitators or by teachers, vary from brief interventions to ones lasting several years (23, 37, 39) and target several outcomes, although most are designed to promote mental wellbeing (40). There has been a focus on individual as opposed to population impact (mean effect as opposed to prevalence), meaning that significant population impacts may have been missed, as well as an underappreciation of the heterogeneity in the school population, meaning that significant sub-group effects may also have been missed (17). The underlying mechanisms that account for the effectiveness of SBMIs in promoting pupil’s mental health are poorly understood (41). Impact evaluations have tended to focus on outcomes with more limited research on potential mediators and moderators, including changes in social relationships and the school/classroom climate (34, 42–44). The potential of whole school (school integrated) interventions to change the school’s culture to a more holistic mindful school, promoting ethical conduct and compassion, and recognizing that mindfulness is a social practice has only recently begun to be explored (27, 34, 45–49).

Most notably, there is a lack of a theoretical base for SBMIs, a rationale for why the intervention should work (50, 51), or a theory of how mindfulness programs work – what mechanisms interventions trigger that facilitate (or hinder/block) the intervention having the intended impact (51, 52). Most notably, the evidence does not indicate *what makes these programs work, how, where, with whom, and to what extent* (26, 37, 53, 54). Most theories assume that practicing mindfulness exercises bring about the observed changes (51, 54, 55) and that mindfulness is about changing the wiring of the brain and the psychology of individuals (55–57). However, constant conjunction does not explain how mindfulness works, and psychological theories of mindfulness are inadequate for explaining how interventions designed to promote mental wellbeing work. Little attention has been paid to changes in teacher-pupil, pupil-pupil or child–parent relationships, or classroom or school climate changes. There is a lack of qualitative (experiential) research on pupils’ (or teachers’) experiences of SBMIs, and little attention has been paid to pupil perspectives on learning mindfulness, how they use mindfulness techniques or what changes they have experienced in their lives (27, 31).

There have been calls for the next generation of research to address these weaknesses (33, 34, 36). These calls have stressed the importance of (1) interdisciplinarity, (2) having a shared definition of mindfulness (38, 58), (3) taking a developmental perspective, shifting from a focus on changes in individual pupils to taking account of the influence of time, social relationships, social interaction and cultural settings and how they may moderate and/or mediate the impact of SBMIs on pupils’ mental wellbeing (27, 43, 49, 59, 60). The importance of understanding how SBMIs work by opening up the ‘black box’ is also acknowledged, and examining the impact of differences in mindfulness interventions (content and delivery) and cultural and socioeconomic context, as well as individual differences including age, gender, healthy status, and ethnicity (21, 27, 34, 61–65). The importance of understanding pupils’ (and their teachers’ and parents’) experience of mindfulness, their agency in response to the intervention and how they have used intervention mechanisms to improve their wellbeing have also been identified as gaps in the existing evidence base (27).

Given the limitations of existing evidence, our synthesis will contribute to the ‘next generation’ of research by generating interdisciplinary transferable knowledge on how SBMIs work and providing guidance on the essential elements required for the successful design, implementation, and uptake of SBMIs. We will go beyond providing evidence on their effects by developing an understanding of the generative mechanisms that lead to outcomes. We will achieve this through refining an *a priori* initial program theory on *how* and *why* universal SBMIs do (or do not) promote pupils’ mental wellbeing. Our primary motivation for undertaking this review is to inform the program theory for SBMIs we are developing to pilot in Rwanda and Ethiopia (66). However, the findings will generate the knowledge to understand better what needs to be done to develop and implement effective and sustainable SBMIs that can be applied in different school contexts more broadly so they benefit pupils’ mental wellbeing and improve their quality of life.

## Aims and objectives

The main objective is to understand how and why universal SBMIs do or do not work, that is, to answer explanatory questions (67).

The aims are to:

- map the evidence on the effectiveness of SBMIs in promoting the wellbeing of children and adolescents attending school;
- describe plausible explanations for the effectiveness of mindfulness interventions designed to promote pupils’ mental wellbeing and
- create transferable theories of how mindfulness interventions promote pupils’ mental wellbeing that can inform program design and implementation in different settings.

To achieve the aims, the objectives are to identify:

- the current evidence on the effectiveness of universal SBMIs in promoting the wellbeing of children and adolescents attending school;
- theories about how mindfulness interventions work in schools;
- the contexts and mechanisms that may facilitate or hinder implementation;
- how pupils and teachers respond to mindfulness interventions (agency);
- how school contexts influence the agency of pupils in responding to the mindfulness intervention and trigger mechanisms that change the context and lead to outcomes;
- how the school system changes (roles and relationships), including pupil-teacher relations and pupil-pupil-relations;
- how the school attitudes and values change (culture) and;
- the outcomes resulting from the interventions.

## Methodology

### Introduction

We will conduct a critical realist synthesis to identify how SBMIs promote pupils’ mental wellbeing. There is no agreed standard or guide for critical realist synthesis reviews, but a critical realist meta-theory underpins them. The design has taken into account recommendations for traditional systematic reviews and narrative synthesis (68–70). PRISMA offers transparency, validity, replicability, and updateability (Supplementary material 2).

A critical realist synthesis is appropriate for our purpose as it enables us to understand how SBMIs promote pupils’ wellbeing what mechanisms the mindfulness training triggers that changes pupils’ agency and improves their mental wellbeing. The objective of other types of systematic reviews does not include doing this. Systematic reviews, meta-analyses, and umbrella reviews aim to provide an evidence-based summary of the evidence of how effective they are. They assume that the outcomes of trials result from a succession of observable events triggered by an intervention. They cannot go beyond demonstrating the relationship between the hypothesized cause and the hypothesized effect (71, 72). Scooping reviews’ primary aims are to provide a mapping of the literature and the identification of gaps in scholarly knowledge.

### A critical realist research paradigm

The design of our proposed synthesis has been informed by the emerging literature proposing and reporting on critical realist synthesis (7, 71, 73–77) and, most notably, building on the approach developed by Sam Porter and his colleagues (78–81) (see Supplementary material 3 for definitions of critical realist terminology). Critical realist synthesis is explanatory; it seeks to explain how interventions work and generate different outcomes in different contexts (82, 83). We will explore how mindfulness interventions are supported or inhibited by contextual mechanisms in schools, how pupils and teachers respond to them and the outcomes that result from the interaction between the intervention and contextual mechanisms and the response of pupils and teachers. In doing so, we will identify the ‘demi-regularities’, the contexts over time and space in which mindfulness interventions enable pupils’ agency to trigger generative mechanisms that promote their mental wellbeing (84). Critical realism seeks to identify, by retroduction and retrodiction, the middle-range interdisciplinary theories that most comprehensively explain how mindfulness interventions work. Such theories are always open to refinement in the light of new evidence.

Critical realism is ontologically realist, arguing that phenomena exist in the social world relatively independent of what we know or think about them, and epistemologically interpretivist, arguing that the way we come to know about phenomena is context-dependent, fallible, prone to individual interpretation, and seen from our angle of vision (67, 85). It rejects Hume’s contention that if B is constantly observed to follow A, then this provides empirical evidence of a direct causal link between the two. It adds value to the realist synthesis practice proposed by the Realist and Meta-Narrative Evidence Stands Project (86) in several ways (77, 87). Firstly, it uses critical realist principles and focuses on developing and testing theories of change to understand what works for whom and under what circumstances. Secondly, it recognizes that the relationship between structure-agent and agent-agent are the fundamental generative mechanisms (7, 73–75, 77). Realist evaluation, however, conflates structure and agency because Pawson and Tilley conceive of social mechanisms as consisting of agency and structure, meaning that the intervention is seen as the main agent of change. Thirdly, critical realism rejects linear, non-reciprocal models of change and emphasizes the complexity of change in social systems involving complex interactions and positive and negative feedback loops (7, 75). Fourthly, critical realism recognizes that all interventions are introduced into open systems, meaning that interventions can work differently in different contexts and the same context over time. Fifthly, critical realism disputes the categorical disconnection between facts and values advanced by Pawson and Tilley and argues that social research should be emancipatory and evaluate the consequences of the outcomes of interventions for those whose lives are impacted by them (77).

**Figure 1:**
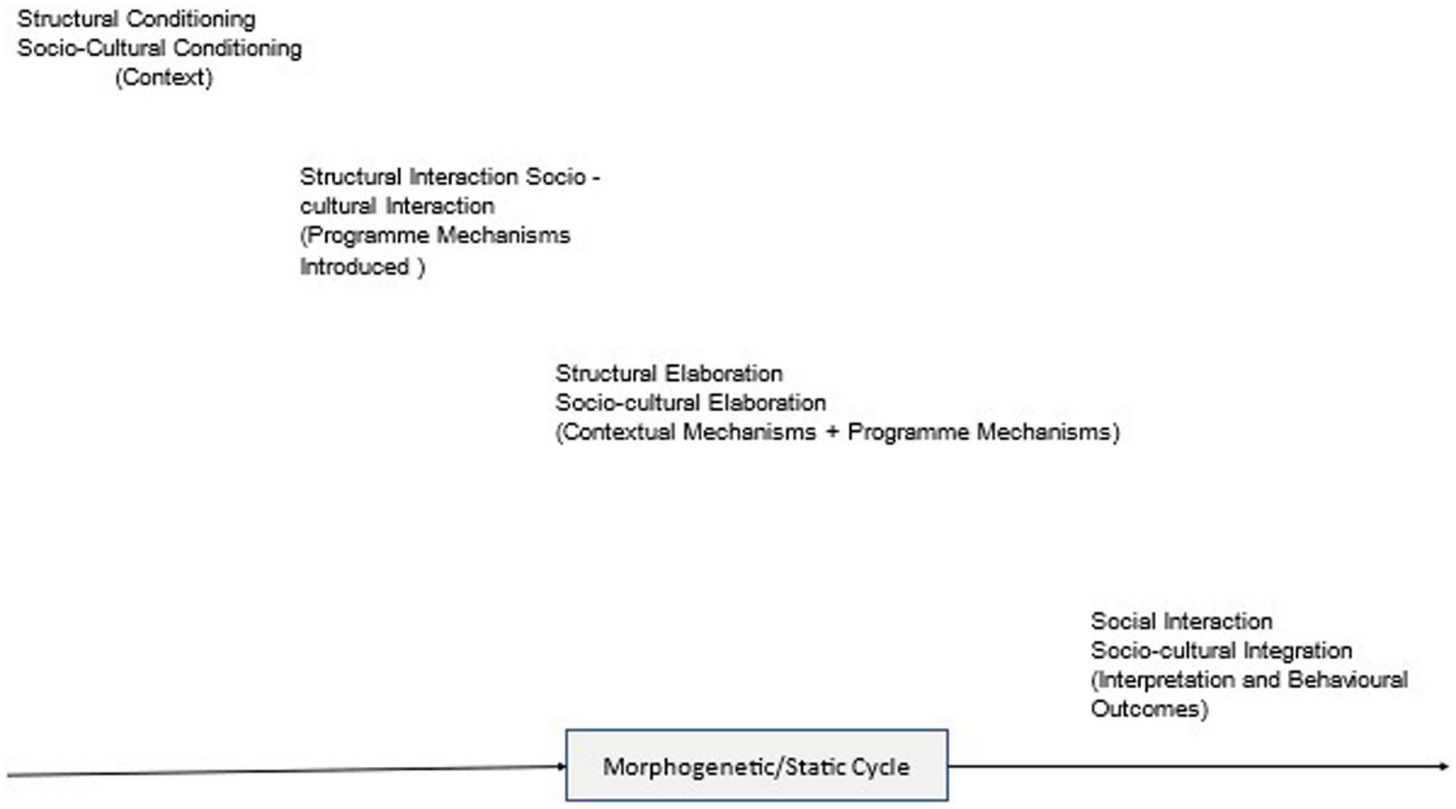
Intervention cycle showing context, agency, intervention, mechanisms outcome (CAIMO) configuration.

Our approach draws on Margaret Archer’s practical morphogenic approach (88, 89), a methodology that complements Critical Realism’s social ontology (90). Archer’s morphogenic approach is the methodological complement to Bhaskar’s model of social transformation (89). She argues that every theory about the social involves understanding the relationship between structure, agency, and culture (SAC) (Figure 1). She argues against conflating agency with structure; the context in which we live comprises both structural and cultural mechanisms, and these mechanisms are analytically separable from and necessarily predate the agency which reproduces or transforms them. Structural and cultural mechanisms are real; they exist independently of individuals and place limitations on opportunities for agency for particular agents at a given time in a given place. The context in which agents live shapes their beliefs, desires, and options and limits their agency – context conditioning. However, the interaction between context mechanisms and agencyshapes and reshapes the context over time; agency can change the context (morphogenesis) or reproduce it (morphostasis). There are three morphogenetic cycles: material, cultural and agency. An intervention such as introducing mindfulness into a school aims to bring about change by giving pupils and teachers the resources to trigger mechanisms that can lead to material and cultural change. Their responses to the intervention are shaped, but not determined, by the context mechanisms, and individuals generate outcomes through actions and interactions. When actors trigger new mechanisms (material and/or cultural), the context changes, but pre-existing context mechanisms can also limit or block social change. Pupils and teachers can resist, redefine, repudiate, suspend, or circumvent engagement with the intervention. Thus, the outcomes of the same intervention can be different in different contexts. Furthermore, while the new social configuration places limitations on agency, it will be subject to agents’ activities, and these will result in its reproduction or transformation.

### Methods/design

The design of the proposed review is based on the five steps used by Mukumbang et al. (7), and we use the critical realist stages of analysis for applied interdisciplinary research: Resolution, Redescription, Retrodiction, Elimination, Identification, Correction (RRREIc) (Table 1) (8, 9). We use the first three stages to develop our initial program theory and then refine and develop it using the six steps of the RRREICc framework to develop theories of how SBMIs work to promote pupils’ mental wellbeing.

**Table 1:** RRREIc stages for interdisciplinary research.

Planning phase	Disciplinary phase	Transdisciplinary phase		Interdisciplinary phase	
Resolution	Redescription	Retrodiction	Elimination	Identification	Refinement
Identification of the levels that are included. Biological/Individual Social- Interaction Socioeconomic	Identification of disciplines and using abduction to develop theoretical explanations for the impact of the intervention on children’s mental wellbeing	Identify how different mechanisms reinforce, moderate, and condition one another to affect the outcome	Elimination of alternative theoretical explanations.	Identify the most comprehensive interdisciplinary explanation for how mindfulness promotes children’s mental wellbeing.	
Identification of disciplines that research at different levels	Develop disciplinary theories to explain the impact. Reductionist/atomistic explanations	Develop trans-factual theories integrating the disciplinary explanations.	Use judgmental rationality to eliminate theories that lack explanatory power.	Use judgmental rationality to identify the most comprehensive explanation	Iterative refinement of theory

#### Step 1: defining the scope of the work and framework formulation

The first step is to define the essential components of a universal SBMI and develop a conceptual model, a theory of how SBMIs work to promote pupils’ wellbeing that we will refine based on the findings from our critical realist synthesis (7, 91). We know that SBMIs work in some contexts. There is evidence that over time and space, there is a relatively enduring relationship between SBMIs and the promotion of pupils’ mental wellbeing, what Tony Lawson refers to as demi regularities (92, 93). In other words, there is a probability that a SBMI will promote pupils’ mental wellbeing. The challenge is to identify under what conditions they do it and how they work to promote pupils’ mental wellbeing. SBMIs are not just complicated; they are complex. They are delivered in schools globally; they involve multiple causal strands and different causal mechanisms operating in other contexts (94). The pathway of change is non-linear, causality is recursive with complex feedback loops, and there are emergent outcomes; that is, mechanisms combine to create something new that cannot be reduced to the mechanisms from which it emerged (7, 75).

For the purposes of this review: (1) mindfulness must be the core component of the intervention; (2) the intervention must be aimed at promoting pupils’ mental wellbeing; (3) delivered during the regular school day, and (4) taught by trained classroom teachers or external facilitators (see Supplementary material 4 for more details of SBMIs).

Mindfulness is a natural state and a disposition. Individual mindfulness as a natural state is intentionally paying attention in a particular way ‘on purpose, in the present moment and non-judgmentally’ (95). Mindfulness has two interrelated dimensions. Firstly, the self-regulation of attention, the conscious monitoring of ongoing subjective experience without distraction or forgetfulness. Secondly, a balanced mental attitude taking a curious, open-minded, and non-reactive orientation toward experiences that naturally arise during daily life. Dispositional and state mindfulness are educable skills that can be developed through sustained practice and cultivated through various mindfulness practices (96, 97). SBMIs fall into three main categories: mindfulness-based stress reduction, mindfulness-based cognitive therapy for children and mindfulness-based Social–Emotional learning (21).

Mental wellbeing can be summed up as feeling good and functioning well as an individual and a member of society (98). It is the outcome of biopsychosocial factors (9, 99) and is dynamic, relational, on a continuum, and occurs within a culture, place and time. Positive wellbeing (flourishing) is multidimensional and includes eudaemonic (positive functioning - seeking meaning, personal growth, and self-realization), hedonic (positive feelings - pursuing pleasurable experiences and positive emotions) (100–102) and harmonic wellbeing (contentment, inner peace, harmony and balance) (103). Elevated levels of wellbeing are associated with positive outcomes, including improved learning, productivity and creativity, good relations, prosocial behavior and good health, and life expectancy.

**Figure 2:**
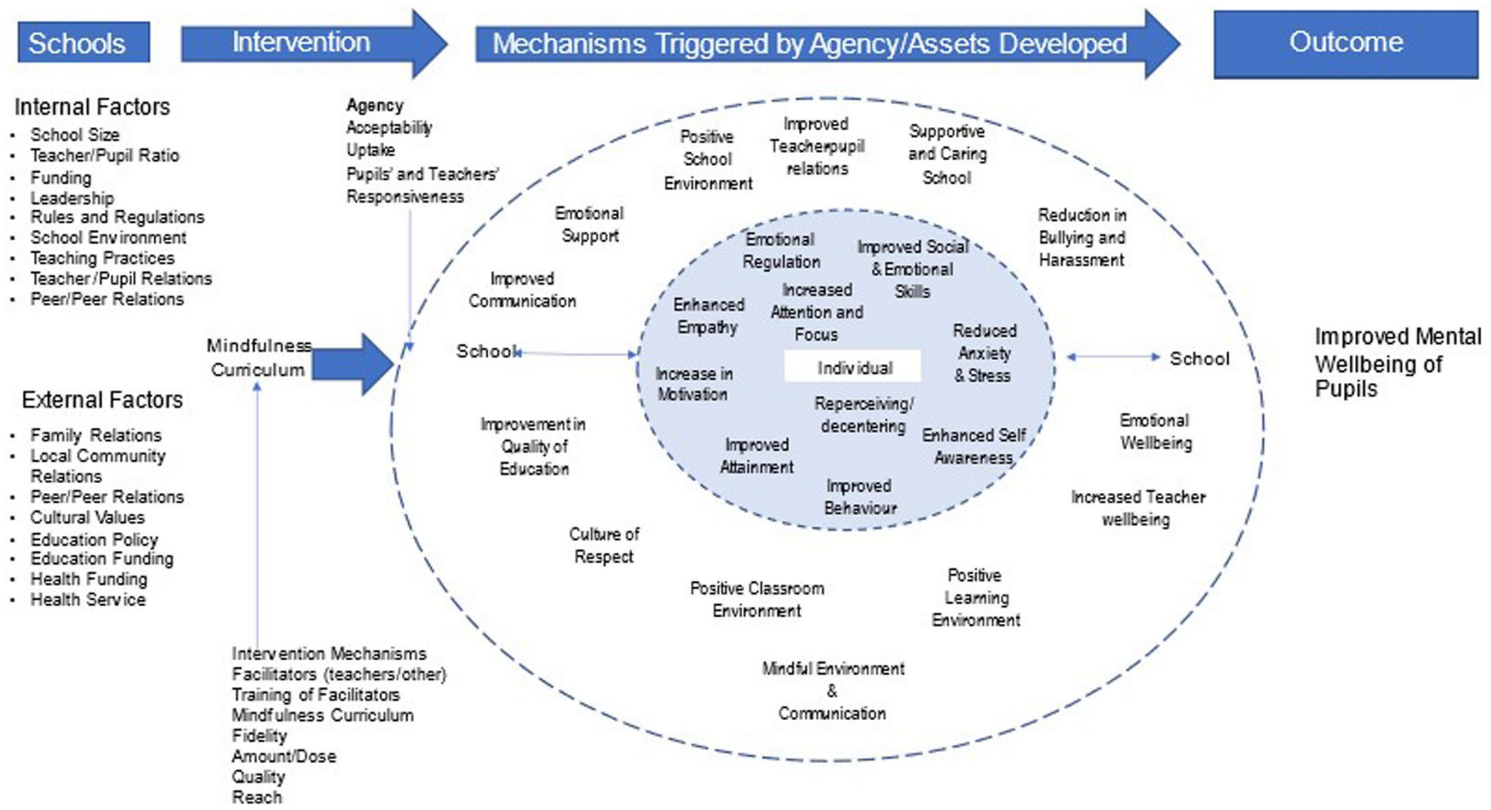
Programme theory.

Our starting point is an initial conceptual model/program theory drawing on systematic reviews of SBMIs and other purposively identified literature to identify ideas and assumptions about how a mindfulness intervention is supposed to work (see Supplementary material 4 for more detail). Informed by Margaret Archer’s Morphogenic (CAIMO) approach and our interdisciplinary perspective, we used abductive thinking and reproductive theorizing to develop our initial program theory (Figure 2). This initial theory will be refined and developed as the review progresses. We hypothesize that SBMIs will have a positive impact on pupils’ mental wellbeing through triggering mechanisms that increase their teachers’ abilities to cope with adversity and improve the school climate (77) and that this will provide a context in which pupils can improve their school performance and enjoy an improved subjective quality of life (56, 83, 84). The complex interaction and reinforcement through feedback loops between the context mechanisms (structural and cultural), actors’ agency and the intervention mechanisms will promote CAs’ mental wellbeing (79, 84–88). However, we remain open to positive, negative, intended, and emergent outcomes.

#### Step 2: search for evidence

##### Search techniques

A systematic Preferred Reporting Items for Systematic Reviews and Meta-Analysis (PRISMA) approach will guide the initial literature search (104). A PRISMA diagram will show the steps of the inclusion and exclusion of documents for the initial search (Supplementary material 5). However, the search will be less formulaic and more iterative than conventional reviews involving multiple search strategies and approaches (7).

The initial literature search will be in three phases: searching electronic databases, searching other sources such as relevant journals and core publishers, and hand searches of relevant review articles, edited book collections and documents identified for data extraction to ensure all relevant studies are included. The aim is to have as wide a range as possible of academic and grey literature with limited restrictions on study type or publication date. No location restrictions will be applied to gain a wide range of relevant studies internationally in high-, middle- and low-income countries. Databases that index health, psychology, sociology and/or education literature will be searched. The search terms and databases used are based on the advice of an academic librarian and our preliminary reading. Searches will be carried out in May 2023.

Twenty-one scholarly databases will be searched: MEDLINE, PsycINFO, Web of Science Core Collection, PubMed Central, Google Scholar, Cochrane Library, ProQuest, SciELO Citation Index, Embase, Sociological Abstracts, Scopus, Current Content Connected, Child Development and Adolescent Studies, British Education Index, Data Citation Index (Clarivate), CINAHL, ERIC, Education Abstracts, Education Research Abstracts, IngentaConnect, and JSTOR.

Eight databases will be used to search for the grey literature: the Bielefeld Academic Search Engine, OpenGrey, PsycEXTRA, ProQuest Dissertations and Theses, Grey Matters, ResearchGate, Academia.edu, and the Social Science Research Network.

For books and book chapters, the websites of eight academic publishers will be searched, Routledge (Taylor & Francis), Palgrave Macmillan, Springer, Elsevier, Wiley, SAGE, Oxford University Press and Cambridge University Press.

The Search terms will be: ‘mindfulness’ or ‘mindful’ or ‘mindfulness-based cognitive therapy’ or MBCT or MBSR and ‘school’ or ‘whole school’ or ‘school-based’ or ‘educational context’ and ‘children’ or ‘adolescents’ or ‘youth’ or ‘young people’ or’ juvenile’ or ‘teen’ or’ young adult’ or ‘teenager’ or ‘pupils.’ We will use Boolean modifiers (Table 2 provides examples of the search terms used for IngentaConnect and Scopus).

**Table 2:** Search terms for IngentaConnect and scopus.

	
**IngentaConnect:**	Article for (Title, Keywords or Abstract contains ‘mindfulness OR mindful OR “mindfulness-based cognitive therapy” OR MBCT OR MBSR AND school OR “whole school” OR “school-based” OR “educational context” AND children OR youth OR “young people” OR juvenile OR teen OR “young adult” OR teenager’) Then, due to last entry not fitting, second search of: article for (Title, Keywords or Abstract contains ‘mindfulness OR mindful OR “mindfulness-based cognitive therapy” OR MBCT OR MBSR AND school OR “whole school” OR “school-based” OR “educational context” AND children OR youth OR “young people” OR juvenile OR teen OR “young adult” OR pupils’)
**Scopus**	Search within Article title, Abstract, Keywords: mindfulness OR mindful OR “mindfulness-based cognitive therapy” OR MBCT OR MBSR AND school OR “whole school” OR “school-based” OR “educational context” AND children OR adolescents OR youth OR “young people” OR juvenile OR teen OR “young adult” OR “teenager” OR pupils

##### Inclusion/exclusion criteria

Exploratory searches suggest that our systematic searches will identify different types of literature that could potentially provide relevant information for our synthesis:

1. Impact evaluations of SBMIs;
2. Literature reviews, including scoping reviews, systematic reviews and meta-analyses/narrative syntheses;
3. Papers reporting on aspects of SMMIs, such as qualitative research with a sample of students who have participated in a SBMI or teachers who have delivered one;
4. Theory papers and books discussing why mindfulness is expected to promote pupils’ mental wellbeing and/or how mindfulness interventions work to produce the outcomes they do.
5. Articles, papers, and books discussing mindfulness in schools more generally.

The inclusion criteria are influenced by the primary purpose of the review, which is to inform the design and delivery of a universal (whole school) SBMI in Rwanda and Ethiopia primary schools. Most pupils attending the schools will be between 7 and 14 years old. The main inclusion criteria will be that the document will likely contribute to answering our research questions/refining our program theory. There will be no time restriction or restriction by geographical location. The included papers will be in English to ensure the researchers can easily understand and interpret them.

The primary source of literature will be papers reporting on impact evaluations of SBMIs. The inclusion criteria for these will be (1) peer-reviewed articles, PhD thesis or a chapter in an edited collection published by a reputable publisher; (2) universal SBMIs; (3) mindfulness is the core educational component of the intervention; (4) the intervention is delivered universally in a mainstream school to at least a whole class; (5) one of the outcome measures is the impact the SBMI has on promoting (aspects of) pupils’ mental wellbeing; (6) pupils aged 7–14 years are among those that participated in the intervention; (7) pre and post-test data are reported. The exclusion criteria will include (1) studies where mindfulness is only one dimension of a program (e.g., Dialectical Behavior Therapy, Commitment Therapy) or the program focusing on yoga, creativity, or other approaches not specific to mindfulness; (2) pupils have explicitly been recruited based on targeted emotional, learning, or behavioral difficulties; (3) only pupils under 7 years or over 14 years participated in the intervention.

The inclusion criteria for systematic reviews, scoping studies, literature reviews and other papers reporting on SBMIs will be (1) that they are published in a peer review journal, as a chapter in an edited collection published by a reputable publisher or a PhD thesis; (2) that they only include universal SBMIs; (3) pupils aged 7–14 years participated in the interventions.

The inclusion criteria for all other documents are that they are (1) relevant, (2) can contribute to the testing of the initial program theory, (3) trustworthy, (4) coherent, and (5) rigorous (105).

##### Article screening

We will screen documents for inclusion using *Covidence* software to manage article screening and data extraction as follows:

1. Remove duplicates and citations without abstracts or summaries;
2. Two reviewers will review the titles and abstracts of all retrieved documents captured by our search strategy and code them as ‘potentially relevant’ and ‘not relevant’. Any disagreements will be resolved by discussion or, if necessary, bringing in a third reviewer;
3. Download the full text of potentially relevant documents and review them against the inclusion and exclusion criteria. A second reviewer will check any documents rejected, and disagreements will be resolved through discussion and, if necessary, bringing in a third reviewer;
4. The reason for the exclusion of any full-text documents will be agreed upon.
5. The downloaded documents will be divided into those reporting on the evaluation of SBMIs and other documents.

#### Step 3: document appraisal and data extraction

Data extraction will progress in two stages. In the first stage, we will extract data from all documents reporting on the findings of SBMIs that meet our inclusion criteria. In the second stage, we will code and extract verbatim data from included documents relevant to the synthesis using the Intervention-context-actor-mechanisms (ICAM) Analytic Tool (see Supplementary material 4: Figure S4.1).

In stage one, we will document differences between SBMIs delivered in different socioeconomic contexts within and across countries, underpinned by various theories, aims, approaches and techniques and delivered differently for different lengths of time (37, 106). The review will also capture other individual differences and program characteristics that can impact program receptivity, including who delivered the training, age of the pupils, gender, reach, quality of delivery, fidelity, responsiveness and mindfulness practice (37, 39, 107). The data will be extracted into an Excel spreadsheet (see Table 3).

**Table 3:** Headings for Stage 1 extraction table.

Document details – title, authors, year of publication, location of study; Country, income group (low, lower-middle, upper-middle, high) and main religion(s); The mindfulness intervention description – the type of SBMI, if manualised, trainers, if classroom-teachers length of training, design, aim/purpose, dose, if homework/practice required; Reach of SBMI class(es), year(s), whole school Sample characteristics - age of pupils, sex/gender, socioeconomic status, ethnicity, type of school (primary/elementary, secondary/high school, public /private); The study design and if it is fit for purpose (quality/rigor); The rationale for SBMI, including any social justice framing; Contextual factors (mechanisms) before the intervention was introduced; Implementation mechanisms – fidelity, dose, quality, and reach Proximal outcomes measured, e.g., attention and concentration, stress and anxiety, emotional regulation, self-awareness, interpersonal skills, behavior problems, reactivity; How proximal outcomes were measured; Distal outcomes measured, e.g., mental health, stress, academic performance, resilience, coping mechanisms; How distal outcomes were measured; Proximal and distal outcomes for teachers; Moderators and mediators, including mindfulness skills; Agency, the responsiveness of pupils and teachers to the intervention and their awareness of its implications for their behavior and experiences; Generative mechanisms triggered by the intervention that could have supported change (positive mechanisms); Generative mechanisms triggered by the intervention that could have restricted/prevented change (negative mechanisms); Contextual mechanisms that could have supported change (positive mechanisms); Contextual mechanisms that restricted/prevented change (negative mechanisms); Summary of main findings. Any theoretical explanations used to explain the outcomes and the level of the explanation, that is, biological, psychological, interpersonal, or structural/cultural; Changes in context following the introduction of the mindfulness intervention; Relevance to the research questions and if the document should be included in the synthesis; Additional notes/comments.

The extraction tool will be piloted. The team will independently read four documents and complete the extraction table. They will then meet, compare their extraction tables, and agree on necessary modifications. Two members of the team will then extract all information. Modified extraction tables will be used to extract information from the literature reviews, theory, and other documents with information relevant to achieving our research aims and objectives.

We will provide a descriptive narrative summary of the findings from this review stage to map the findings from research on universal SBMIs comprehensively. This will provide an understanding of what works, that is the effectiveness of universal SBMIs across different contexts, using different interventions and for different groups of pupils.

We will review and refine our inclusion and exclusion criteria and conduct further searches before undertaking the critical realist analysis, where we are concerned with understanding (theorizing) how SBMIs work. We will search for other evidence where we have insufficient evidence of findings to support the synthesis and remove any documents containing insufficient relevant data to inform the synthesis (identifying the underlying causal mechanisms and context that explain program outcomes and provide insight into how mindfulness programs can be successfully implemented in or transferred to other contexts). We will use the Research Evidence Extraction/appraisal Tool (Supplementary material 6) and remove any document not using credible and trustworthy methods. Any documents identified as not contributing and/or not using credible and reliable methods will be reviewed by a second reviewer, with differences being resolved by discussion and, if necessary, by bringing in a third reviewer. The reasons for the exclusion of any document will be noted. Two reviewers will review any documents we identify by targeted searches and, if included, the reason for the inclusion noted.

We will then extract the data relevant for the synthesis review and code and organize the abstracted data against the four critical realist evaluation categories: contextual mechanisms, intervention mechanisms, human agency, and outcomes. We are extracting data that will enable us to test our realist hypothesis about the rules, resources and norms embedded in the intervention and its context and their relationship; this will mainly be qualitative (process) research findings and the findings from structural equation modeling of the pathways from the intervention to the outcomes (72, 108–110). Qualitative research findings enable us to uncover how pupils (and their teachers) participating in mindfulness interventions interpret and respond to and are impacted by the intervention.

#### Step 4: synthesize evidence and draw conclusions

The analysis will follow the critical realist stages of analysis for applied interdisciplinary research (RRREIc) (Table 1 above) (8, 9). The framework consists of six steps to build transdisciplinary accounts of phenomena. These are: (1) break down complex events into component parts and identify disciplinary explanations of impact; (2) redescribe impacts in mono-theoretically meaningful ways; (3) use retrodiction to identify trans-factual theories that explain the ways the mechanisms interact to produce outcomes; (4) eliminate alternative competing hypotheses; (5) identify the most comprehensive explanation; (6) refine scientific knowledge in light of (provisional) findings.

**Figure 3:**
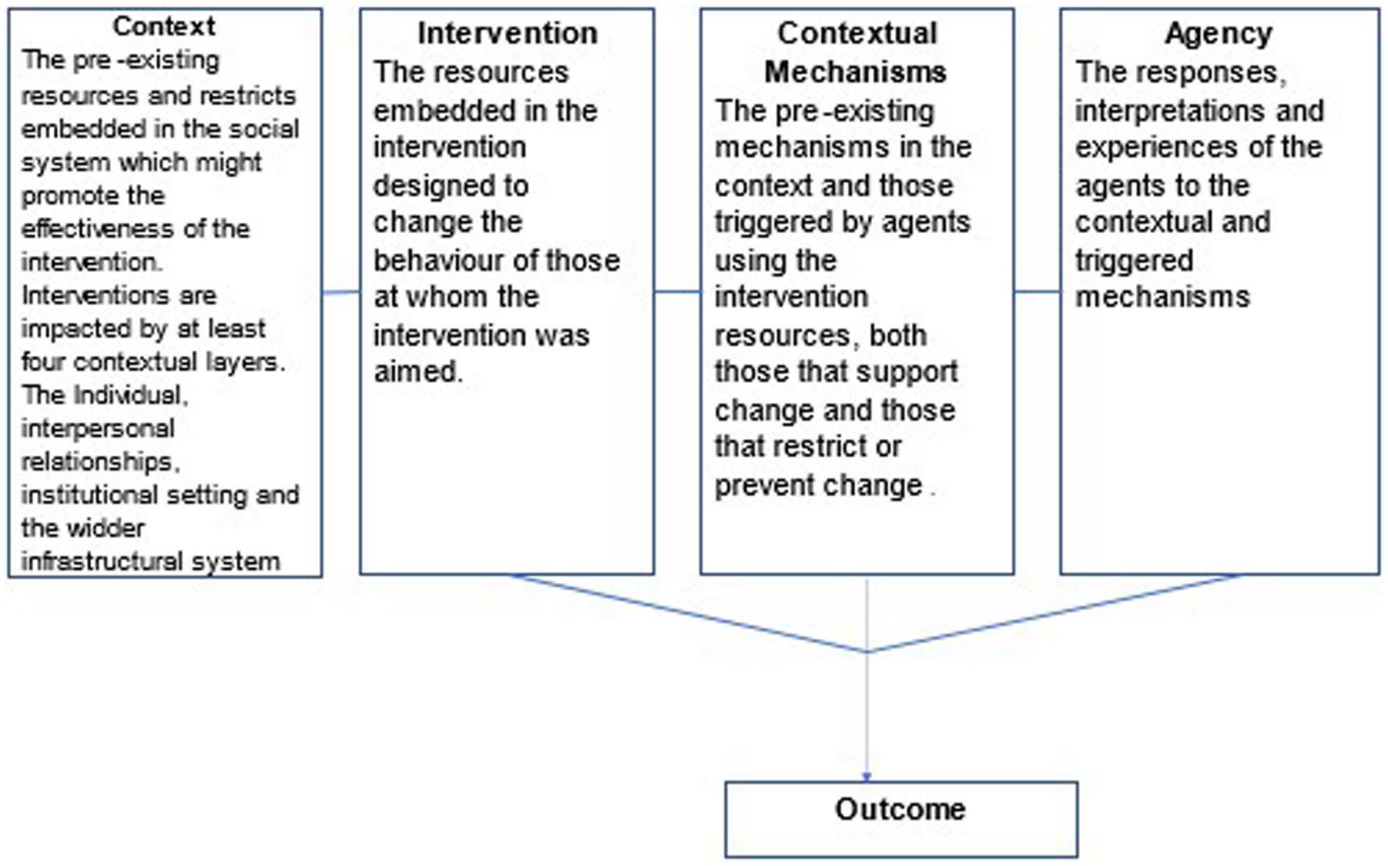
Critical realist evaluation process.

To do this, we will first systematically build configurations of intervention, context, agency mechanism, and outcome (ICAMO) for each mechanism identified (111). The four critical realist evaluation categories are the context (social structure and culture) before and as it changes during the intervention, pupils’ (and teachers’ agency), the mechanisms triggered by pupils’ (and teacher’s) agency and the outcomes. Agency is pupils’ (and teachers’) responses to the mindfulness interventions; mechanisms are triggered by agency and enable pupils (and teachers) to benefit from the intervention by changing the pre-existing context conditions (social structures and culture). The contextual conditions can facilitate or inhibit pupils’ potential to benefit from the intervention. Outcomes are the mental well-being benefits pupils, gain from participating in universal SBMIs. Within each category, findings will be broken down thematically and reported narratively to distinguish between different contexts, agency responses, mechanisms triggered, and outcomes (Figure 3). The key themes that describe processes and causal mechanisms for explaining mindfulness intervention outcomes in schools will then be identified. Hypothetical links will then be made between the ICAMO themes, creating potential pathways that account for the impacts of school-based mindfulness interventions on pupils and why, for whom and under what circumstances these impacts occur.

**Figure 4:**
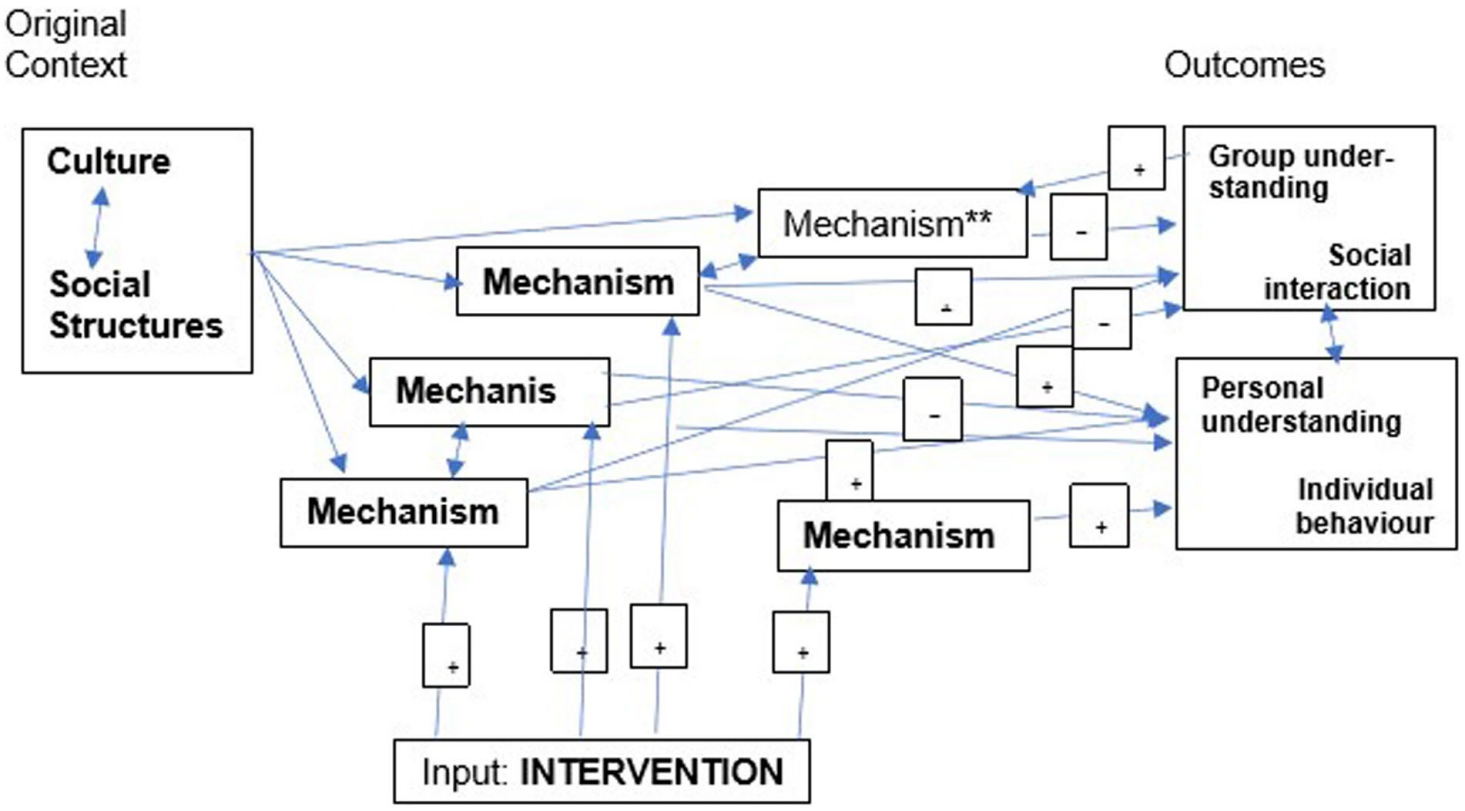
Simplified hypothetical causal pathway of change after an intervention.

However, to take account of complexity, it may be necessary to develop a non-linear pathway of change showing how the complex interaction of mechanisms (context mechanisms that predate the intervention and those triggered by the intervention) leads to the observed outcomes (Figure 4) (7, 74, 75). To do this, we will use feedback loop diagrams to model change, showing both mechanisms triggered by the intervention that cause change, those already in the context that supported change (+ve mechanism) and those already in the context and mechanisms triggered by the intervention that restricted or prevented change (−ve mechanisms). Outcomes will likely be more complex than a dichotomy between morphogenesis (structural change) and morphostasis (structural reproduction).

#### Step 5: disseminate and evaluate

We will publish at least one article in a peer-reviewed journal reporting the findings from the literature review, conforming to RAMESES publication standards (112) and a policy brief intended for policymakers with the target audience including WHO, UNESCO, UNICEF and the UK and Scottish Governments. Findings from the review will be disseminated via an article in the Conversation, seminar and conference presentations, and podcasts posted on the project website and disseminated by social media.

##### Patient and public involvement

The development of this protocol involved no patients or other participants, as it will draw on existing research studies. The project, of which the proposed literature review is an element, has community and pupil reference groups and teacher and policy actor involvement in designing and delivering the mindfulness intervention. We will discuss the findings from this review with these groups.

## Discussion

Our review will be the first critical realist (or realist) review of the literature on mindfulness interventions in schools. As well as informing the program theory for the main study, it will enable policymakers and school leaders to understand under what circumstances school-based mindfulness interventions promote pupils’ mental wellbeing and for which pupils they work.

Adopting an interdisciplinary approach will enable us to add to the dominant psychological one by providing an understanding of how the political, economic and cultural contexts within and outside of schools can contribute to the outcomes, an understanding of how mindfulness interventions are perceived and accepted among diverse groups of students; a consideration of structural influences on the outcomes of mindfulness interventions at the institutional, community and country levels; a recognition of the importance of social class, gender, ethnicity, age and other protected characteristics, on differential outcomes on mindfulness interventions; and a consideration of pedagogical strategies, such as whole school interventions that integrate mindfulness into the everyday routine of the school, interventions where mindfulness is taught as a stand-alone course for a limited number of weeks.

The main challenges are likely to be that most studies will have been carried out in high-income countries, so we will not be able to identify critical context elements and relevant mechanisms that are unique to low-and-middle-income countries; there may be little information in the documents on the initial context; and the documents may not include details of the theoretical reasoning underpinning the intervention. There is a risk of bias because we will only include materials published in English, thereby omitting potentially relevant studies in other languages. There is less risk of publication bias due to evaluations of interventions that did not have a positive outcome being less likely to be published than those that do because we are mainly interested in explaining how interventions worked.

From our reading of the literature for developing the program theory, we are aware that differences by age (especially between children and adolescents), gender, socioeconomic status, the type of intervention and who delivers it (teachers or external facilitators), among other differences are potentially significant for understanding why some interventions work and others do not. We included extracting data for these characteristics in our table (Table 3). However, we also know that not all articles have all this information, which may limit our analysis.

## Open access

This is an open-access article distributed under the terms of the Creative Commons Attribution License, which permits unrestricted use, distribution, and reproduction in any medium, provided the original author and source are credited.

## Author contributions

PA: Conceptualization, Funding acquisition, Methodology, Writing – original draft, Writing – review & editing. GN: Funding acquisition, Writing – review & editing. IS: Project administration, Writing – review & editing. LD’A: Funding acquisition, Writing – review & editing.

## References

[1] Fusar-Poli P Correll CU Arango C Berk M Patel V Ioannidis JPA. Preventive psychiatry: a blueprint for improving the mental health of young people. World Psychiatry. (2021) 20:200–21. doi: 10.1002/wps.20869, PMID: 34002494 PMC8129854

[2] Oliver C . Critical realist grounded theory: a new approach for social work research. Br J Soc Work. (2012) 42:371–87. doi: 10.1093/bjsw/bcr064

[3] Bhaskar R . Scientific realism and human emancipation. Abingdon: Routledge (2009).

[4] Goldhagen J Clarke A Dixon P Guerreiro AI Lansdown G Vaghri Z. Thirtieth anniversary of the UN convention on the rights of the child: advancing a child rights-based approach to child health and well-being. BMJ Paediatr Open. (2020) 4:1–8. doi: 10.1136/bmjpo-2019-000589, PMID: 32099906 PMC7015043

[5] Lotz-Sisitka H . What’s in a conference theme? Some reflections on critical realist research and its emergence in Africa over a period of 20+ years. J Crit Realis. (2022) 21:483–501. doi: 10.1080/14767430.2023.2146923

[6] Eastwood JG Jalaludin BB Kemp LA. Realist explanatory theory building method for social epidemiology: a protocol for a mixed methods multilevel study of Neighbourhood context and postnatal depression. Springerplus. (2014) 3:1–12. doi: 10.1186/2193-1801-3-12, PMID: 24422187 PMC3888492

[7] Mukumbang FC De Souza D Liu H Uribe G Moore C Fotheringham P . Unpacking the design, implementation and uptake of community-integrated health care services: a critical realist synthesis. BMJ Glob Heal. (2022) 7:1–14. doi: 10.1136/bmjgh-2022-009129, PMID: 35940630 PMC9364400

[8] Bhaskar R . Contexts of interdisciplinarity Interdisciplinarity and climate change In: Bhaskar R Frank C Høyer KG Parker P Næss J, editors. Interdisciplinarity and climate change transforming knowledge and practice for our global future. Abingdon and New York, NY: Routledge (2010)

[9] Bhaskar R Danermark B Price L. Interdisciplinarity and wellbeing: A critical realist general theory of Interdisciplinarity. London and New York, NY: Routledge (2018).

[10] Pulimeno M Piscitelli P Colazzo S Colao A Miani A. School as ideal setting to promote health and wellbeing among young people. Heal Promot Perspect. (2020) 10:316–24. doi: 10.34172/hpp.2020.50, PMID: 33312927 PMC7723000

[11] Patel V Rahman A. An agenda for global child mental health. Child Adolesc Ment Health. (2015) 20:3–4. doi: 10.1111/camh.12083, PMID: 32680324

[12] O’Reilly M Svirydzenka N Adams S Dogra N. Review of mental health promotion interventions in schools. Soc Psychiatry Psychiatr Epidemiol. (2018) 53:647–62. doi: 10.1007/s00127-018-1530-1, PMID: 29752493 PMC6003977

[13] McDaid D Park A-L Wahlbeck K. The economic case for the prevention of mental illness. Annu Rev Public Health. (2019) 40:373–89. doi: 10.1146/annurev-publhealth-040617-013629 30601725

[14] Huppert FA Johnson DM. A controlled trial of mindfulness training in schools: the importance of practice for an impact on well-being. J Posit Psychol. (2010) 5:264–74. doi: 10.1080/17439761003794148

[15] Rose G . Sick individuals and sick populations. Int J Epidemiol. (2001) 30:427–32. doi: 10.1093/ije/30.3.427 11416056

[16] Kuyken W Nuthall E Byford S Crane C Dalgleish T Ford T . The effectiveness and cost-effectiveness of a mindfulness training programme in schools compared with normal school provision (MYRIAD): study protocol for a randomised controlled trial. Trials. (2017) 18:194. doi: 10.1186/s13063-017-1917-4, PMID: 28446223 PMC5406917

[17] Greenberg MT Abenavoli R. Universal interventions: fully exploring their impacts and potential to produce population-level impacts. J Res Educ Eff. (2017) 10:40–67. doi: 10.1080/19345747.2016.1246632

[18] Kessler RC Angermeyer M Anthony JC De GRD Demyttenaere K Gasquet I . Lifetime prevalence and age-of-onset distributions of mental disorders in the World Health Organization’s world mental health survey initiative. World Psychiatry. (2007) 6:168–76. PMID: 18188442 PMC2174588

[19] Bährer-kohler S Carod-Artal FJ. Global mental health: Prevention and promotion. Cham: Springer (2017).

[20] Kieling C Baker-Henningham H Belfer M Conti G Ertem I Omigbodun O . Child and adolescent mental health worldwide: evidence for action. Lancet. (2011) 378:1515–25. doi: 10.1016/S0140-6736(11)60827-1, PMID: 22008427

[21] Hosan NE Smith V Strean WB Sibinga EMS Punja S Vohra S. The “what,” “why,” and “when” of using mindfulness in schools: best practices and guidance for educators and policymakers. Theory Pract. (2022) 61:465–76. doi: 10.1080/00405841.2022.2107822

[22] Ergas O Hadar LL. Mindfulness in and as education: a map of a developing academic discourse from 2002 to 2017. Rev Educ. (2019) 7:757–97. doi: 10.1002/REV3.3169

[23] Schonert-Reichl K Roeser RW. Handbook of mindfulness in education: Integrating theory and research into practice. Cham: Springer (2016).

[24] McCabe M Costello S Roodenburg J. The child’s voice in determining program acceptability for a school-based mindfulness intervention. Soc Sci. (2017) 6:155. doi: 10.3390/socsci6040155

[25] Thomas G Atkinson C. Perspectives on a whole class mindfulness programme. Educ Psychol Pract. (2017) 33:231–48. doi: 10.1080/02667363.2017.1292396

[26] Hutchinson JK Huws JC Dorjee D. Exploring experiences of children in applying a school-based mindfulness programme to their lives. J Child Fam Stud. (2018) 27, 3935–3951. doi: 10.1007/s10826-018-1221-2

[27] Sheinman N Hadar LL Gafni D Milman M. Preliminary investigation of whole-school mindfulness in education programs and children’s mindfulness-based coping strategies. J Child Fam Stud. (2018) 27:3316–28. doi: 10.1007/s10826-018-1156-7

[28] Ager K Albrecht NJ Cohen M. Mindfulness in schools research project: exploring students’ perspectives of mindfulness. Psychology. (2015) 6:896–914. doi: 10.4236/psych.2015.67088

[29] Dariotis JK Mirabal-Beltran R Cluxton-Keller F Gould LF Greenberg MT Mendelson T. A qualitative evaluation of student learning and skills use in a school-based mindfulness and yoga program. Mindfulness (N Y). (2016) 7:76–89. doi: 10.4049/jimmunol.1801473.The, PMID: 26918064 PMC4762597

[30] Viafora DP Mathiesen SG Unsworth SJ. Teaching mindfulness to middle school students and homeless youth in school classrooms. J Child Fam Stud. (2015) 24:1179–91. doi: 10.1007/s10826-014-9926-3

[31] Sapthiang S Van Gordon W Shonin E. Health school-based mindfulness interventions for improving mental health: a systematic review and thematic synthesis of qualitative studies. J Child Fam Stud. (2019) 28:2650–8. doi: 10.1007/s10826-019-01482-w

[32] Montero-Marin J Allwood M Ball S Crane C De Wilde K Hinze V . School-based mindfulness training in early adolescence: what works, for whom and how in the MYRIAD trial? Evid Based Ment Health. (2022) 25:117–24. doi: 10.1136/ebmental-2022-300439, PMID: 35820993 PMC9340034

[33] Roeser RW Schussler D Baelen RN Galla BM. Mindfulness for students in pre-K to secondary school settings: current findings, future directions. Mindfulness. (2023) *14* :233–8. doi: 10.1007/s12671-022-02036-w

[34] Roeser RW Greenberg MT Frazier T Galla BM Semenov AD Warren MT. Beyond all splits: envisioning the next generation of science on mindfulness and compassion in schools for students. Mindfulness (N Y). (2022) 14:239–54. doi: 10.1007/s12671-022-02017-z PMC11784944 39897709

[35] Emerson LM de Diaz NN Sherwood A Waters A Farrell L. Mindfulness interventions in schools: integrity and feasibility of implementation. Int J Behav Dev. (2020) 44:62–75. doi: 10.1177/0165025419866906

[36] Schonert-Reichl K . Encouraging advances in the science on mindfulness and compassion in schools: current research, lingering questions, and future directions. Mindfulness. (2023) *14* :300–6. doi: 10.1007/s12671-023-02070-2

[37] Semple RJ Droutman V Reid BA. Mindfulness Goes to school: things learned (So far) from research and real-world experiences. Psychol Sch. (2017) 54:29–52. doi: 10.1002/pits.21981, PMID: 28458403 PMC5405439

[38] Crane RS Brewer J Feldman C Kabat-Zinn J Santorelli S Williams JMG . What defines mindfulness-based programs? The warp and the weft. Psychol Med. (2017) 47:990–9. doi: 10.1017/S0033291716003317, PMID: 28031068

[39] Carsley D Khoury B Heath NL. Effectiveness of mindfulness interventions for mental health in schools: a comprehensive Meta-analysis. Mindfulness (N Y). (2018) 9:693–707. doi: 10.1007/s12671-017-0839-2

[40] Phan ML Renshaw TL Caramanico J Greeson JM MacKenzie E Atkinson-Diaz Z . Mindfulness-based school interventions: a systematic review of outcome evidence quality by study design. Mindfulness (N Y). (2022) 13:1591–613. doi: 10.1007/s12671-022-01885-9 36186722 PMC9524483

[41] der Gucht K Van TK Kuppens P Raes F. Potential moderators of the effects of a school-based mindfulness program on symptoms of depression in adolescents. Mindfulness (N Y). (2017) 8:797–806. doi: 10.1007/s12671-016-0658-x

[42] Monsillion J Zebdi R Romo-desprez L. School mindfulness-based interventions for youth, and considerations for anxiety, depression, and a positive school climate—a systematic literature review. Children. (2023) 10:861. doi: 10.3390/children10050861, PMID: 37238409 PMC10217750

[43] Dai X Du N Shi S Lu S. Effects of mindfulness-based interventions on peer relationships of children and adolescents: a systematic review and Meta-analysis. Mindfulness (N Y). (2022) 13:2653–75. doi: 10.1007/s12671-022-01966-9

[44] Meiklejohn J Phillips C Freedman ML Griffin ML Biegel G Roach A . Integrating mindfulness training into K-12 education: fostering the resilience of teachers and students. Mindfulness (N Y). (2012) 3:291–307. doi: 10.1007/s12671-012-0094-5

[45] Sapthiang S Van Gordon W Shonin E. Mindfulness in schools: a health promotion approach to improving adolescent mental health. Int J Ment Health Addict. (2019) 17:112–9. doi: 10.1007/s11469-018-0001-y

[46] Tarrasch R . Mindful schooling: better attention regulation among elementary school children who practice mindfulness as part of their school policy. J Cogn Enhanc. (2017) 1:84–95. doi: 10.1007/s41465-017-0024-5

[47] Ergas O . Mindfulness in, as and of education: three roles of mindfulness in education. J Philos Educ. (2019) 53:340–58. doi: 10.1111/1467-9752.12349

[48] Brito R Joseph S Sellman E. From instrumental to integral mindfulness: toward a more holistic and transformative approach in schools. Stud Philos Educ. (2022) 41:91–109. doi: 10.1007/s11217-021-09810-8

[49] Callen-Davies RJ Bristow J Gazder T Griffith GM Noorani Y Crane RS. Mindfulness-based programmes and ‘bigger than self’ issues: protocol for a scoping review. BMJ Open. (2023) 13:e067819. doi: 10.1136/bmjopen-2022-067819, PMID: 36921950 PMC10030572

[50] Shute RH . Schools, mindfulness, and metacognition: a view from developmental psychology. Int J Sch Educ Psychol. (2019) 7:123–36. doi: 10.1080/21683603.2018.1435322

[51] Shute RH . School-based mindfulness interventions. Oxford Res Encyclopedia Educ. (2019). doi: 10.1093/acrefore/9780190264093.013.979

[52] Phipps A . Mindfulness in schools: exploring the impact on internalising difficulties, the role of home practice and the mechanisms of psychological change. London: University College London: (2016).

[53] Ditrich T . Theorising mindfulness: conceptualisations and research In: Ditrich T Wiles R Lovegrove B, editors. Mindfulness and education research and practice. Newcastle upon Tyne: Cambridge Scholars Publishing. (2017). Available at: https://www.cambridgescholars.com/product/978-1-4438-1688-5

[54] Laurenzi CA Mamutse S Marlow M Mawoyo T Stansert Katzen L Carvajal-Velez L . Critical life course interventions for children and adolescents to promote mental health. Cambridge Prism Glob Ment Heal. (2023) 10:e4. doi: 10.1017/gmh.2022.58, PMID: 36843881 PMC9947636

[55] Micklitz K Wong G Howick J. Mindfulness-based programmes to reduce stress and enhance well-being at work: a realist review. BMJ Open. (2021) 11:1–16. doi: 10.1136/bmjopen-2020-043525, PMID: 33741667 PMC7986896

[56] Teasdale J . What happens in mindfulness. New York, NY and London: Guilford Press (2022).

[57] Forbes D . Mindfulness and its discontents: Education, self, and social transformation. New York, NY: Fernwood Publishing (2019).

[58] Felver JC Cary EL Helminen EC Schutt MKA Gould LF Greenberg MT . Identifying Core program components of mindfulness-based programming for youth: Delphi approach consensus outcomes. Mindfulness (N Y). (2023) 14:279–92. doi: 10.1007/s12671-022-02015-1

[59] Khoury B Grégoire S Dionne F. The interpersonal dimension of mindfulness. Ann Med Psychol (Paris). (2020) 178:239–44. doi: 10.1016/j.amp.2018.10.018

[60] Berger R Brenick A Tarrasch R. Reducing Israeli-Jewish pupils’ outgroup prejudice with a mindfulness and compassion-based social-emotional program. Mindfulness (N Y). (2018) 9:1768–79. doi: 10.1007/s12671-018-0919-y

[61] Kuyken W Ball S Crane C Ganguli P Jones B Montero-Marin J . Effectiveness and cost-effectiveness of universal school-based mindfulness training compared with normal school provision in reducing risk of mental health problems and promoting well-being in adolescence: the MYRIAD cluster randomised controlled trial. Evid Based Ment Health. (2022) 25:99–109. doi: 10.1136/ebmental-2021-300396, PMID: 35820992 PMC9340028

[62] Dunning D Tudor K Radley L Dalrymple N Funk J Vainre M . Do mindfulness-based programmes improve the cognitive skills, behaviour and mental health of children and adolescents? An updated meta-analysis of randomised controlled trials. Evid Based Ment Health. (2022) 25:135–42. doi: 10.1136/ebmental-2022-300464, PMID: 35820989 PMC9340039

[63] Filipe MG Magalhães S Veloso AS Costa AF Ribeiro L Araújo P . Exploring the effects of meditation techniques used by mindfulness-based programs on the cognitive, social-emotional, and academic skills of children: a systematic review. Front Psychol. (2021) 12. doi: 10.3389/fpsyg.2021.660650 PMC8632731 34867573

[64] Baelen RN Gould LF Felver JC Schussler DL Greenberg MT. Implementation reporting recommendations for school-based mindfulness programs. Mindfulness. (2022) *14* :255–78. doi: 10.1007/s12671-022-01997-2

[65] Andreu CI García-Rubio C. How does mindfulness work in schools? An integrative model of the outcomes and mechanisms of change of mindfulness-based interventions in the classroom In: Steinebach C Langer AI, editors. Enhancing resilience in youth. Cham: Springer (2019). 139–57. doi: 10.1007/978-3-030-25513-8

[66] Abbott P D’Ambruoso L Yared M McNamee P Hailu T Nzabalirwa W. A critical realist informed pilot cluster control trial evaluating the effectiveness of a mindfulness intervention for promoting child and adolescent mental wellbeing in Rwanda and Ethiopia. Res Regist. (2023):8799.

[67] Sayer A . Method in social science: A realist approach. 2nd ed. London: Routledge (2010).

[68] Gough D Oliver S Thomas J. Learning from research: Systematic reviews for informing policy decisions: A Quick Guide. In: A paper for the Alliance for Useful Evidence. London: Nesta. (2013). Available at: https://apo.org.au/sites/default/files/resource-files/2013-12/apo-nid71119.pdf

[69] Moher D Liberati A Tetzlaff J Altman DGG. Preferred reporting items for systematic reviews and Meta-analyses: the PRISMA statement. Ann Intern Med. (2009) 151:264–9. doi: 10.7326/0003-4819-151-4-200908180-00135 19622511

[70] Lockwood C Munn Z Porritt K. Qualitative research synthesis: methodological guidance for systematic reviewers sutilising meta-aggregation. Int J Evid Based Healthc. (2015) 13:179–87. doi: 10.1097/XEB.0000000000000062 26262565

[71] Porter S McConnell T Reid J. The possibility of critical realist randomised controlled trials. Trials. (2017) 18:133. doi: 10.1186/s13063-017-1855-1, PMID: 28327182 PMC5359862

[72] Singleton H Porter S Beavis J Falconera L Hernandez JP Holley D. Accounting for complexity in critical realist trials: the promise of PLS-SEM. J Crit Realis. (2023) 22:384–403. doi: 10.1080/14767430.2023.2217652

[73] De Souza DE . Critical realism and realist review: sAnalysing complexity in educational restructuring and the limits of generalizing program theories across Borders. Am J Eval. (2016) 37:216–37. doi: 10.1177/1098214015605175

[74] Bellazzecca E Teasdale S Biosca O Skelton DA. The health impacts of place-based creative programmes on older adults’ health: a critical realist review. Heal Place. (2022) 76:102839. doi: 10.1016/j.healthplace.2022.102839, PMID: 35691142

[75] Hinds K Dickson K. Realist synthesis: a critique and an alternative. J Crit Realis. (2021) 20:1–17. doi: 10.1080/14767430.2020.1860425

[76] Brannan MJ Fleetwood S O’Mahoney J Vincent S. Critical essay: Meta-analysis: a critical realist critique and alternative. Hum Relations. (2017) 70:11–39. doi: 10.1177/0018726716674063

[77] Porter S . The uncritical realism of realist evaluation. Evaluation. (2015) 21:65–82. doi: 10.1177/1356389014566134

[78] Spacey A Porter S. Understanding advance care planning in care homes throughout the COVID-19 pandemic: a critical realist review and synthesis. Palliat Med. (2022) 37:663–76. doi: 10.1177/02692163221137103, PMID: 36373288 PMC9659704

[79] Porter S . Realist evaluation: An immanent critique. Nurs Philos. (2015) 16:239–51. doi: 10.1111/nup.12100, PMID: 26392234

[80] Spacey A Scammell J Board M Porter S. A critical realist evaluation of advance care planning in care homes. J Adv Nurs. (2021) 77:2774–84. doi: 10.1111/jan.14822, PMID: 33751625

[81] Spacey A Scammell J Board M Porter S. Systematic critical realist review of interventions designed to improve end-of-life care in care homes. Nurs Health Sci. (2020) 22:343–54. doi: 10.1111/nhs.12665, PMID: 31797527

[82] Clegg S . Evidence-based practice in educational research: a critical realist critique of systematic review. Br J Sociol Educ. (2005) 26:415–28. doi: 10.1080/01425690500128932

[83] Mingers J Standing C. Why things happen – developing the critical realist view of causal mechanisms. Inf Organ. (2017) 27:171–89. doi: 10.1016/j.infoandorg.2017.07.001

[84] Lawson T . Economics and reality. London: Routledge (1997).

[85] Danermark B Ekström M Karlsson JC. Explaining society: Critical realism in the social sciences. London and New York, NY: Routledge (2019).

[86] Greenhalgh T Wong G Westhorp G Pawson R. Protocol - realist and meta-narrative evidence synthesis: evolving standards (RAMESES). BMC Med Res Methodol. (2011) 11:Article 115. doi: 10.1186/1471-2288-11-115, PMID: 21843376 PMC3173389

[87] Mukumbang FC De Souza DE Eastwood JG. The contributions of scientific realism and critical realism to realist evaluation. J Crit Realis. (2023) 22:504–24. doi: 10.1080/14767430.2023.2217052

[88] Archer M . Culture and agency: The place of culture in social theory. 2nd ed. Cambridge: Cambridge University Press (1998).

[89] Archer M . Realist social theory: The morphogenetic approach. Cambridge: Cambridge University Press (2008).

[90] Archer M . The morphogenetic approach: critical Realism’s explanatory framework approach In: Róna P Zsolnai L, editors. Agency and causal explanation in economics. Cham: Springer (2020)

[91] Eastwood JG Kemp LA Garg P Tyler I De Souza DE. A critical realist translational social epidemiology protocol for concretising and contextualising a theory of neighbourhood context, stress, depression, and the developmental origins of health and disease (DOHaD), Sydney Australia. Int J Integr Care. (2019) 19:8–13. doi: 10.5334/ijic.3962, PMID: 31367207 PMC6659581

[92] Lawson T . Economic science without experimentation In: Archer M Bhaskar R Collier A Lawson T Norrie A, editors. Critical realism: essential readings. London: Routledge (1988)

[93] Næss P . ‘Demi-regs’, probabilism and partly closed systems. J Crit Realis. (2019) 18:475–86. doi: 10.1080/14767430.2019.1644951

[94] Rogers PJ . Using programme theory to evaluate complicated and complex aspects of interventions. Evaluation. (2008) 14:29–48. doi: 10.1177/1356389007084674

[95] Kabat-Zinn J . Wherever you go, there you are: Mindfulness meditation in everyday life. New York: Hyperion (1994).

[96] Daniel C Walsh I Mesmer-Magnus J. Mindfulness: unpacking its three shades and illuminating integrative ways to understand the construct. Int J Manag Rev. (2022) 24:654–83. doi: 10.1111/ijmr.12296

[97] Roeser RW Galla BM Baelen RN. Mindfulness in schools: evidence on the impacts of school-based mindfulness programs on student outcomes in P–12 educational settings. A Policy Brief for Robert Wood Johnston Foundation (2022) Available at: https://prevention.psu.edu/wp-content/uploads/2022/09/PSU-Mindfullness-Brief-2022.pdf

[98] WHO . Mental health. (2022) Availabel at: https://www.who.int/news-room/fact-sheets/detail/mental-health-strengthening-our-response (Accessed October 5, 2023)

[99] Pilgrim D . Some implications of critical realism for mental health research. Soc Theory Heal. (2014) 12:1–21. doi: 10.1057/sth.2013.17

[100] Huppert FA So TTC. Flourishing across Europe: application of a new conceptual framework for defining well-being. Soc Indic Res. (2013) 110:837–61. doi: 10.1007/s11205-011-9966-7, PMID: 23329863 PMC3545194

[101] Diener E Wirtz D Tov W Kim-Prieto C Choi D Oishi S . New well-being measures: short scales to assess flourishing and positive and negative feelings. Soc Indic Res. (2010) 97:143–56. doi: 10.1007/s11205-009-9493-y

[102] Ruggeri K Garcia-Garzon E Maguire Á Matz S Huppert F. Well-being is more than happiness and life satisfaction: a multidimensional analysis of 21 countries. Health Qual Life Outcomes. (2020) 18:1–16. 32560725 10.1186/s12955-020-01423-y PMC7304199

[103] Gallup and the Wellbeing for, Planet Earth Foundation . Wellbeing for all: Incorporating harmonic principles of wellbeing in subjective wellbeing research and policymaking. (2023) Available at: https://www.gallup.com/analytics/510770/inclusive-wellbeing-research.aspx

[104] Page MJ Moher D Bossuyt PM Boutron I Hoffmann TC Mulrow CD . PRISMA 2020 explanation and elaboration: updated guidance and exemplars for reporting systematic reviews. BMJ. (2021) 372:n160. doi: 10.1136/bmj.n160, PMID: 33781993 PMC8005925

[105] Dada S Dalkin S Gilmore B Hunter R Mukumbang FC. Applying and reporting relevance, richness and rigour in realist evidence appraisals: advancing key concepts in realist reviews. Res Synth Methods. (2023) 14:504–14. doi: 10.1002/jrsm.1630, PMID: 36872619

[106] Hart R Ivtzan I Hart D. Mind the gap in mindfulness research: a comparative account of the leading schools of thought. Rev Gen Psychol. (2013) 17:453–66. doi: 10.1037/a0035212

[107] Tudor K Maloney S Raja A Baer R Blakemore SJ Byford S . Universal mindfulness training in schools for adolescents: a scoping review and conceptual model of moderators, mediators, and implementation factors. Prev Sci. (2022) 23:934–53. doi: 10.1007/s11121-022-01361-9, PMID: 35267177 PMC9343282

[108] Brown A Hecker KG Bok H Ellaway RH. Strange bedfellows: exploring methodological intersections between realist inquiry and structural equation modeling. J Mix Methods Res. (2021) 15:485–506. doi: 10.1177/1558689820970692

[109] Osteen PJ . A critical realist exploration of the relationship between personal and professional value Systems in Social Workers and the impact on motivations for participation in a social work community of practice. University of Denver (2009). Available at: https://digitalcommons.du.edu/cgi/viewcontent.cgi?article=1896&context=etd

[110] Pratschke J . Realistic models? Critical realism and statistical models in the social sciences. Philosophica. (2003) 71:13–38. doi: 10.21825/philosophica.82236

[111] Mukumbang FC Marchal B Van Belle S van Wyk B. Using the realist interview approach to maintain theoretical awareness in realist studies. Qual Res. (2020) 20:485–515. doi: 10.1177/1468794119881985

[112] Wong G Westhorp G Manzano A Greenhalgh J Jagosh J Greenhalgh T. RAMESES II reporting standards for realist evaluations. BMC Med. (2016) 14:96–18. doi: 10.1186/s12916-016-0643-1, PMID: 27342217 PMC4920991

[113] Abbott P D’Ambruoso L Yared M McNamee P Nzabalirwa W. Study protocol for a critical realist pilot cluster-randomised control trial of a whole-school-based mindfulness intervention (SBMI) promoting child and adolescent mental wellbeing in Rwanda and Ethiopia. medRxiv. (2023). doi: 10.1101/2023.05.10.23289769

