# Supplementary material for: A protocol for a critical realist synthesis of school mindfulness interventions designed to promote pupils’ mental wellbeing
Source: Front Public Health. 2024 Jan 9;11:1309649. doi: 10.3389/fpubh.2023.1309649 (PMC10803664; doi:10.3389/fpubh.2023.1309649)
Supplement: Supplementary file 1 [file Data_Sheet_1.PDF]

Supplementary Material 1

Table S1.1a: Findings from Systematic Reviews of School-Based Mindfulness Interventions[i]

Authors and Year	Number of Articles Reviewed	Age Range of Children	Review Topic/Objective	Main findings
(Monsillion et al., 2023)	12<br>Universal<br>Total sample 2042	5-18 years	Anxiety, depression, and positive school climate	1. Positive outcomes for emotional and behavioural regulation, prosocial behaviours and reducing stress.<br>2. MBIs potentially a mediator in improving students wellbeing and the school/class climate.
(Pickerell et al., 2023)	18 MBIs<br>1 targeted, 17 universal<br><br>Total sample 3406	7-12 years	Emotional regulation	A significant improvement in emotional awareness, an increase in positive emotions and a reduction in depression.
(Dai et al., 2022)	21<br>18 in schools of which 8 targeted<br>2179 participants	5-18 years	Effect of MBIs on peer relationships	1. Small positive impact on peer relationships.<br>2. Main moderators were participants age and facilitators background. Among pupils the largest effect was amongst adolescents and interventions that were facilitated by a combination of teachers and external facilitators.<br>3. The outcome did not vary by other moderators such as gender, preexisting clinical conditions, intervention dosage or research design.
(Phan et al., 2022)	77<br>No breakdown by targeted and universal<br><br>Total sample of 12,358 students	Kindergarten to 12th Grade	All outcomes as targeted by the studies included.	1. Grade A evidence[ii]: increase in prosocial behaviour, resilience, executive functioning, attention, and mindfulness.<br>Decrease in anxiety, attention problems/ADHD behaviours and conduct behaviours.<br>2. Grade B evidence: improved self-concept, social competency, self and emotion regulation, coping, executive functioning, cognitive control and mood,

				social basis, and attention problems. Improved academic performance.<br>3. Smaller group of studies (B grade) suggested positive changes in physiology, neurophysiology, and brain plasticity.
(Roeser et al., 2022)	54<br>No breakdown by targeted and universal<br><br>Total sample 13,000+ pupils	Kindergarten to12th grade	Student outcomes as in the review studies (1) mindfulness and self-regulation skills, (2) mental health, (3) physical health, (4) healthy relationships, (5) school behaviour and performance.[iii]	1. 36 different SBMPs, 30% adapted MBSR/MBCT, 48% novel, 22% brief practices, 50% external facilitator, 32% classroom teacher, 25% less than an hour, 50% one hour to less than 15 hours and 25% more than 15 hours.<br>2. Positive outcome for mindfulness and self-regulatory skills for kindergarten and elementary school pupils, for internalising distress, positive outcome for elementary and secondary school pupils, healthy relationships for all grades, school behaviour for kindergarten and elementary pupils and physical health for secondary school pupils.<br>3. Little evidence that mindfulness interventions have a positive impact on externalising distress or psychological wellbeing.
(Tudor et al., 2022)	31<br>Inclusion criteria included universal interventions.<br><br>Total sample size 7532	11-18 years	Provide an overview of the mediators, moderators and implementation effects on the impact of SBMIs. Quantitative studies.	1. The studies most frequently tested for stress (13), mindfulness (13), depression (12), anxiety (11), self-regulation (12), wellbeing (7), self-compassion (5), weight/shape concerns (4), school/classroom climate (3 studies), substance use (2 studies), resilience (2 studies)<br>2. 11 studies assessed potential moderators these included gender (6 studies), age (2), baseline mental health (4), high attachment anxiety (1), higher agreeableness and lower emotional stability (1), cognitive variables (1), school type (1), pre-treatment -mindfulness. No

			No limit on outcomes	consistency in acting as moderators or in outcomes moderated.<br>3. 5 studies assessed potential mediators of outcomes. One study found that improvements in mindfulness skills mediated a number of mental health and behavioural skills and another that an improvement in mindfulness skills correlated with a decrease in affect disturbance and an increase in positive affect. Another study found that deceases in cognitive reactivity mediated decreases in symptoms of depression, anxiety and stress and another found that changes in school climate did not mediate the relationship between SBMT and improved performance.<br>4. 25 studies reported data relating to implementation: 10 studies reported programme dosage but only one tested the impact of dosage on outcomes and found that higher dosage was related to increased positive attitudes to school and a decrease in disturbance post-intervention.<br>5. 22 studies reported an indicator of participant responsiveness. Only one study explored the association between responsiveness and outcomes, with higher satisfaction with the mindfulness intervention was associated with pre-post test improvements in affective self-regulation efficacy and emotional awareness.<br>6. 12 studies measured the extent of student self-reported practice outside of the delivery of the intervention (homework). Most studies reported low levels of practice. Seven studies measured the influence of practice on outcomes. Four of them found a significant

				association between practice and outcomes and three found no significant difference.
(Filipe et al., 2021)	29<br>All universal<br>Total sample size 1874	6-12 years	Review the available evidence about the efficacy of mediation techniques used by mindfulness-based programmes on cognitive, socio-emotional and academic skills.	1. Of the 16 studies that assessed cognitive skills all found significant effects.<br>2. Of the 21 studies that assessed socio-emotional skills 19 found a significant effect.<br>3. Of the three studies that measured academic skill one found a significant effect.<br>4. Range of interventions and variable results across studies.
(Segal et al., 2021)	8 (7 unique samples)<br>2 targeted, 5 universal<br><br>Total sample 1,139	Grades 1-12	Outcomes of interventions in low-income schools	Findings were inconsistent across the studies with some reporting improvements for externalising and internalising symptoms, emotional regulation, and perceived stress.<br>High Enrollment and retention, moderate levels of student reported satisfaction and low adherence to at-home practice.
(Baelen et al., 2019)	34<br>6 targeted<br>28 Universal<br>Total sample 6,271	3-18 years	Evidence for mindfulness training programmes delivered in schools	1. For three studies there was no change on the outcomes of interest.<br>2. For one study there was no change on the outcomes of interest, but home practice was associated with improvements in mindfulness and wellbeing.<br>3. For the remaining 30 studies there was a positive change in at least some of the outcomes of interest.

				4. Positive outcomes included promoting mental health and psychological wellbeing, self-regulation (including executive functioning, behavioural self-regulation, and emotion regulation) and improving school grades.<br>5. Studies often inconsistent in reporting some positive outcomes for elements of wellbeing and not others or reported positive outcomes for some domains and not others.<br>6. Programmes differ in effectiveness for different outcomes and age groups and pupils with higher risks benefit more from universal interventions than others.
(McKeering and Hwang, 2019)	13<br>13 universal<br>Total sample<br>2, 277	11-14 years	Reported outcomes of SBMIs,	1. 11 out of 13 studies reported positive outcomes on different wellbeing variables.<br>2. 9 of the 11 quantitative studies reported positive improvements in physiological, cognitive, and emotional wellbeing.<br>3. In the six studies with qualitative data, pupils were generally positive and active. Pupils reported seeing positive changes in their physical, mental and relational experiences.
(Sapthiang et al., 2019)	7<br>2 targeted, 5 universal<br><br>Total sample size 251	Grades 1-12	Systematic review and thematic synthesis of qualitative evidence on benefits of mindfulness training.	Pupils identified four main benefits of participating in SBMIs,(1) using attentional process to regulate emotions and cognitions, (2) stress reduction, (3) improved coping and social skills and (4) calming and/or relaxation.
(Carsley et al., 2018)	24<br>Breakdown of targeted and	6-18 years	Asses the strength of the effects of	1. Small but positive effect of mindfulness interventions.<br>2. Interventions delivered during late teens had the greatest impact on mental health and wellbeing.

	universal interventions not given.<br><br>Sample of pupils 2179		school-based mindfulness interventions on mental health and wellbeing and to evaluate and compare the effects of these interventions based on, development period, gender, type of mindfulness intervention and the Identity of the facilitator.	3. Females seem to respond better to mindfulness interventions than males.<br>4. Interventions combing various mindfulness activities and yoga-based mindfulness activities had more impact than existing or pre-designed mindfulness programmes.<br>5. Interventions led by a trained teacher more impact mental health than those facilitated by an outsider, while those facilitated by an outsider more impact mindfulness.<br>6. The quality score of the study did not moderate the impact.
(Rawana et al., 2018)	67<br>No breakdown by targeted and universal but includes both.<br><br>Total sample size not given.	4-29 years	Effects of SBMIs on executive functioning, emotional development, clinical mental health and psychological wellbeing	1. Pre-school children experiences improved executive functioning and psychological wellbeing.<br>2. Primary school children experienced improved academic performance and reduced negative effects and improved and executive functioning.<br>3. Most middle school children experienced improved psychological wellbeing, reduced (improved prosocial and positive behaviour and reduced stress), reduced clinical symptoms (improved anxious and depressive symptoms).<br>4. Adolescents (high school) mixed results for psychological wellbeing and reduction in depressive and anxious symptoms.

				5. Post secondary students experienced increased psychological wellbeing, reduced clinical symptoms and improved mindfulness scores.
(Maynard et al., 2017)	61 (44 randomised/quasi-experimental) (35 included in the metanalysis)<br><br>Total sample 6,207 students		The purpose of the review was to examine and synthesize evidence of mindfulness-based interventions implemented in school settings on achievement, behaviour and socioemotional outcomes.	1. Wide variety of mindfulness interventions but most at least partially manualised and of shorter duration.<br>2. Meta analysis found small but significant findings for socioemotional outcomes and cognitive outcomes with the studies producing similar results despite the heterogeneity.<br>3. Limited evidence for introduction of mindfulness in schools. Also no evidence influences behaviour or educational performance.
(Felver et al., 2016)	28<br>11 targeted and 17 universal<br><br>Sample 3414 students	5-17 years	The main aim of this systematic review was to identify limitations in the literature reporting on mindfulness interventions in schools to	1. Range of interventions used including manualised interventions<br>2. Outcomes were varied depending on the dependent variable measures. Studies variously reported a decrease in psychopathology following the intervention (behavioural problems, anxiety, depression, affective disturbances, problems with executive functioning and attention and suicide ideation.<br>3. Studies also frequently reported increases in prosocial behavioural attributes, with the attributes varying widely.

			inform future research.	4. Four studies demonstrated befits to psychological functioning.
(Zenner et al., 2014)	24<br>No breakdown by targeted and universal<br>Sample 1348 students	6-23 years		1. Meta analysis mindfulness has a positive impact on cognitive performance, stress, and resilience.<br>2. 8 studies provided information on acceptability and experience generally seen as positive.<br>3. Heterogeneity of studies.

Table S1.1b: Findings from a Literature Review

**Authors and Year**	**Inclusion Criteria**	**Main Aims of the Review**	**Main findings**
(Hosan et al., 2022)	SBMIs '---- that include assessments of mindfulness, investigate rigorously evaluated MBIs, or are RCTs to ensure that changes in the outcome variables are attributable to mindfulness and not potential confounding variables ---' (467)	To identify studies meeting their inclusion criteria that have proved effective.<br><br>They do not aim to identify all studies but give examples.<br><br>They includes studies with universal, indicated and selective samples and pre-kindergarten to K12	1. 4 studies improved specific mental health concerns and 3 improved pupils' overall sense of wellbeing.<br>2. 6 studies that improved various mental health problems with all the studies reporting that pupils participating in the mindfulness intervention having fewer internalising and externalising symptoms post intervention compared with their peers who did not participate.<br>3. 7 studies improved, wellbeing, executive functioning, and resilience.<br>4. 6 studies increased prosocial behaviours and healthy relationships.<br>5. 5 studies found evidence of improved academic achievement (3), school engagement (1), decrease in maladaptive perfectionism (1) and the school climate (1).<br>6. However, there are also other rigours studies that suggested mixed findings and a lack of evidence for some mindfulness programmes.

Baelen, R. N., Esposito, J. L., and Galla, B. M. (2019). “A selective review of mindfulness training programmes for children and adolescents in school settings,” in *The Mindful School*, ed. P. A. Jennings (New York and London: Guildford Press).

Carsley, D., Khoury, B., and Heath, N. L. (2018). Effectiveness of Mindfulness Interventions for Mental Health in Schools: A Comprehensive Meta-analysis. *Mindfulness (N. Y).* 9, 693–707. doi:10.1007/s12671-017-0839-2.

Dai, X., Du, N., Shi, S., and Lu, S. (2022). Effects of Mindfulness-Based Interventions on Peer Relationships of Children and Adolescents: a Systematic Review and Meta-analysis. *Mindfulness (N. Y).* 13, 2653–2675. doi:10.1007/s12671-022-01966-9.

Felver, J. C., Celis-de Hoyos, C. E., Tezanos, K., and Singh, N. N. (2016). A Systematic Review of Mindfulness-Based Interventions for Youth in School Settings. *Mindfulness (N. Y).* 7, 34–45. doi:10.1007/s12671-015-0389-4.

Filipe, M. G., Magalhães, S., Veloso, A. S., Costa, A. F., Ribeiro, L., Araújo, P., et al. (2021). Exploring the Effects of Meditation Techniques Used by Mindfulness-Based Programs on the Cognitive, Social-Emotional, and Academic Skills of Children: A Systematic Review. *Front. Psychol.* 12. doi:10.3389/fpsyg.2021.660650.

Hosan, N. E., Smith, V., Strean, W. B., Sibinga, E. M. S., Punja, S., and Vohra, S. (2022). The “what,” “why,” and “when” of using mindfulness in schools: Best practices and guidance for educators and policymakers. *Theory Pract.* 61, 465–476. doi:10.1080/00405841.2022.2107822.

Mackenzie, K., and Williams, C. (2018). Universal, school-based interventions to promote mental and emotional wellbeing: What is being done in the UK and does it work? A systematic review. *BMJ Open* 8. doi:10.1136/bmjopen-2018-022560.

Maynard, B. R., Solis, M. R., Miller, V. L., and Brendel, K. E. (2017). Mindfulness-based interventions for improving cognition, academic achievement, behavior, and socioemotional functioning of primary and secondary school students. *Campbell Syst. Rev.* 13, 1–144. doi:10.4073/csr.2017.5.

McKeering, P., and Hwang, Y. S. (2019). A Systematic Review of Mindfulness-Based School Interventions with Early Adolescents. *Mindfulness (N. Y).* 10, 593–610. doi:10.1007/s12671-018-0998-9.

Monsillion, J., Zebdi, R., and Romo-desprez, L. (2023). School Mindfulness-Based Interventions for Youth, and Considerations for Anxiety, Depression, and a Positive School Climate—A Systematic Literature Review. *Children* 10, 861. Available at: https://www.mdpi.com/2227-9067/10/5/861.

Phan, M. L., Renshaw, T. L., Caramanico, J., Greeson, J. M., MacKenzie, E., Atkinson-Diaz, Z., et al. (2022). Mindfulness-Based School Interventions: a Systematic Review of Outcome Evidence Quality by Study Design. *Mindfulness (N. Y).* 13, 1591–1613. doi:10.1007/s12671-022-01885-9.

Pickerell, L. E., Pennington, K., Cartledge, C., Miller, K. A., and Curtis, F. (2023). The Effectiveness of School-Based Mindfulness and Cognitive Behavioural Programmes to Improve Emotional Regulation in 7–12-Year-Olds: A Systematic Review and Meta-Analysis. *Mindfulness (N. Y).*, 1068–1087. doi:10.1007/s12671-023-02131-6.

Rawana, J. S., Diplock, B. D., and Chan, S. (2018). “Mindfulness-Based Programs in School Settings: Current State of the Research,” in *Handbook of School-Based Mental Health Promotion*, eds. A. W. Leschied, G. L. Flett, and D. H. Saklofske (Cham: Springer).

Roeser, R. W., Galla, B. M., and Baelen, R. N. (2022). Mindfulness in Schools: Evidence on the Impacts of School-Based Mindfulness Programs on Student Outcomes in P–12 Educational Settings. A Policy Brief for Robert Wood Johnston Foundation. Available at: https://prevention.psu.edu/wp-content/uploads/2022/09/PSU-Mindfullness-Brief-2022.pdf.

Sapthiang, S., Van Gordon, W., and Shonin, E. (2019). Health School-based Mindfulness Interventions for Improving Mental Health: A Systematic Review and Thematic Synthesis of Qualitative Studies. *J. Child Fam. Stud.* 28, 2650–2658. doi:10.1007/s10826-019-01482-w.

Segal, S. C., Vyas, S. S., and Monson, C. M. (2021). A Systematic Review of Mindfulness-Based Interventions in Low-Income Schools. *Mindfulness (N. Y).* 12, 1316–1331. doi:10.1007/s12671-020-01571-8.

Tudor, K., Maloney, S., Raja, A., Baer, R., Blakemore, S. J., Byford, S., et al. (2022). Universal Mindfulness Training in Schools for Adolescents: a Scoping Review and Conceptual Model of Moderators, Mediators, and Implementation Factors. *Prev. Sci.* 23, 934–953. doi:10.1007/s11121-022-01361-9.

Zenner, C., Herrnleben-Kurz, S., and Walach, H. (2014). Mindfulness-based interventions in schools-a systematic review and meta-analysis. *Front. Psychol.* 5, 1–20. doi:10.3389/fpsyg.2014.00603. PMID: 25071620; PMCID: PMC4075476.

---

[i] Universal interventions are intended for the general population and are generally taught to whole classes or to all the children in a school during the normal school day. They can be taught by classroom teachers, by external mindfulness facilitators, by study researcher/programme author or by a combination. The length of training for teachers for delivering the mindfulness intervention varies from a few days to several weeks. We have included some reviews where not all the studies were universal or in schools but the majority were.

[ii] See Table 1, p 1595 of Phan et al 2023 for details of the grading system. A Grade A indicates strong evidence and a Grade B good evidence based on the quality review of the paper.

[iii] (1) **Mindfulness and self-regulation skills,**

Mindfulness Skills: Mindfulness of sensation, emotion and thought; self-compassion

Attention Regulation: Selective attention; attentional switching; working memory; self-control; inhibitory control

Emotional Regulation: Emotional awareness and processing; impulse control; improved cognitive reappraisal; emotional expression; positive coping; coping self-efficacy.

(2) **Mental health**

Internalizing Distress: Stress, symptoms of anxiety and depression; negative affect; test anxiety; rumination; reactive responses to stress; somatic complaints; negative coping

Externalizing Distress: Impulsivity; hyperactivity and social problems; anger; anger expression/aggression; hostility; attention problems

Psychological Well-Being: Positive affect; resilience; optimism; positive self-concept

(3) **Physical health**

Physiological Indicators: Blood pressure; heart rate; cortisol output (stress hormones); body mass index

Behavioral Indicators: Sleep; mindful eating; health care utilization; intention to use substances and abstention from alcohol use; positive and negative alcohol expectancies; drinking refusal self-efficacy; dietary restraint.

(4) **Healthy relationships**

Prosocial Skills and Altruism: Social skills; socio-emotional competence; empathy; kindness, compassion for others, diminished affective prejudice and stereotyping

Positive Connections to Others: Cooperation, popularity; positive peer and teacher relations; positive social climate I Positive Connections with Nature: Connection with nature and others; sustainable consumption behaviour.

(5) **School behaviour and performance**

Academic Motivation: Academic self-concept, motivation to learn

Academic Behaviors: Classroom engagement, rule-following, lack of disruptive behaviors

Academic Performance: Grades, academic skills (Roeser et al., 2022, 6)
