# Supplementary material for: A protocol for a critical realist synthesis of school mindfulness interventions designed to promote pupils’ mental wellbeing
Source: Front Public Health. 2024 Jan 9;11:1309649. doi: 10.3389/fpubh.2023.1309649 (PMC10803664; doi:10.3389/fpubh.2023.1309649)
Supplement: Supplementary file 2 [file Data_Sheet_2.PDF]

Supplementary Material 2
PRISMA-P 2015 Checklist

**Section and topic**	**Item No**	**Checklist item**	**Page**
**ADMINISTRATIVE INFORMATION**			
Title:			
Identification	1a	The report is identified as a protocol of a critical realist synthesis review	2
Update	1b	The protocol is not an update of a previous systematic review	n/a
Registration	2	The review is registered with PROSPERO	3
Authors:			
Contact	3a	The name, institutional affiliation, and e-mail address of all protocol authors are provided with the physical mailing address of the corresponding author	1
Contributions	3b	The contributions of protocol authors are described	24
Amendments	4	The protocol is not an amendment of a previously completed or published protocol	n/a
Support:			
Sources	5a	Sources of financial support for the review have been identified	Financial disclosure
Sponsor	5b	The review funder and sponsor have been provided	Financial disclosure
Role of sponsor or funder	5c	The role of external support in developing the protocol has been provided	n/a
Rationale	6	The rationale for the review in the context of what is already known is described	3 - 6
Objectives	7	An explicit statement of the question(s) the review will address with reference to participants, interventions, comparators, and outcomes (PICO) is provided	6 - 7
Eligibility criteria	8	The study characteristics to be used as criteria for eligibility for the review are described in accordance with realist review publication standards	14
Information sources	9	All intended information sources are described	12 - 14
Search strategy	10	The search strategy is described	12 - 14
Study records:			

Data management	11a	The tools that will be used to manage records and data throughout the review are described	14
Selection process	11b	The study selection process is described, including the involvement of a second reviewer to corroborate the data extracted and theory generation	15 - 16
Data collection process	11c	The planned method of extracting data from reports is provided	15 -16
Data items	12	The data items relate to the context, agency, intervention, and mechanisms configuration, which has been described in detail	16-18
Outcomes and prioritisation	13	The outcomes for which data will be sought relate to SBMIs as described within the protocol. There is no prioritisation	16 - 18
Risk of bias in individual studies	14	n/a as critical realist reviews consider the relevance and rigour of included studies, this process is described	n/a
Data synthesis	15a	The criteria under which study data will be synthesised are described	18
	15b	n/a data will not be extracted for quantitative synthesis	n/a
	15c	n/a data will not be extracted for additional analyses (such as sensitivity or subgroup analyses, meta-regression)	n/a
	15d	n/a data will not be extracted for quantitative synthesis; therefore, alternative synthesis methods are not appropriate	n/a
Meta-bias(es)	16	The realist review is not considering any planned assessment of meta-bias(es) (such as publication bias across studies, selective reporting within studies)	n/a
Confidence in cumulative evidence	17	The strength of the body of evidence will be determined using a series of judgements from the review authors in alignment with previous critical realist reviews and realist review publication standards	7

Source: Moher D et al.: Preferred reporting items for systematic review and meta-analysis protocols (PRISMA-P) 2015 statement. Systematic Reviews 2015 4:1
