# Supplementary material for: A protocol for a critical realist synthesis of school mindfulness interventions designed to promote pupils’ mental wellbeing
Source: Front Public Health. 2024 Jan 9;11:1309649. doi: 10.3389/fpubh.2023.1309649 (PMC10803664; doi:10.3389/fpubh.2023.1309649)
Supplement: Supplementary file 3 [file Data_Sheet_3.PDF]

Supplementary Material 3
Critical Realist Terminology/Key Tenants and their Ontological/Epistemological Basis

**Agency**	The power of individuals to engage in meaningful actions. Agents have causal powers because they respond to the intervention and, within their context, can trigger mechanisms that change the context.
**Abduction**	Reinterpreting events into a conceptual framework.
**Complexity**	Outcomes result from the complex interaction of generative mechanisms in an open system. Therefore, change is non-linear, emergent and involves feedback loops.
**Context**	The contexts set limits to the efficacy of a programme intervention. 'Structures' are material, physical, and human resources, and associated practices. 'Culture' is the realm of intersubjectivity, social values/ideas, and ideational influences. Contexts are dynamic and can change through agency triggered by the intervention. However, the same causal mechanisms can produce different outcomes in different contexts.
**Context Mechanisms**	The resources and restrictions embedded in the social and organisational (relational) structure that can influence the outcomes and must be identified. They can be physical, social, interpersonal, intrapersonal, or conceptual. They have causal powers and include social structures, cultural structures, institutions, conventions, norms, and values. While they may not be observable, they can be retroduced from what is observed.
**Culture**	Intersubjectivity, ideas and ideational influences.
**Demi-regularities**	Partial event regularities indicate that an intervention in some contexts triggers mechanisms that result in the same outcome.
**Depth Ontology**	Reality is stratified into three domains.<br>1. The Empirical: experiences of what happens;<br>2. The Actual: things that occur independently of whether we observe them or not;<br>3. The Real: which is beyond our direct observation. It consists of the underlying, unobservable, and often hidden structures and mechanisms that shape the actual.
**Emergence**	The ability of generative mechanisms to combine to create something new that cannot be reduced to the generative mechanisms from which it emerged. Complex systems exhibit new properties, patterns and behaviours that arise from the interactions and relationships between these components. The whole is greater than the aggregation of its constituent parts.
**Independent reality**	The world exists independently of our understanding of it.
**Intervention**	The intervention is designed to bring about change in a given context.
**Intervention mechanisms**	The generative mechanisms are the underlaying causal powers triggered by the experiences, interpretations, and responses to interventions by actors that can change the context mechanisms.

**Judgemental Rationalism**	The evaluation of competing explanations to identify the one that has the most credible explanation, the one that is most practically adequate.
**Laminated System**	The social world is understood as an open, complex, stratified (layered) system of objects that make things happen. There are seven layers, from the Global to the sub-individual level, each influencing the other.
**Middle-Range Theory**	Middle-range theories lie between untestable grand theory and concrete description. They are theories that can generate hypotheses that can be tested and refined through empirical research.
**Morphogenic Approach**	Based on the view that there are three primary causal powers in society, structure, culture and agency, and that structure and culture necessarily predate the actions that transform them.
**Open System**	A (social) system where multiple causal factors interact and influence each other, and it is impossible to isolate variables as in laboratory conditions. The multiplicity of mechanisms other than the ones triggered by the intervention means that what happens may not necessarily be what was envisaged.
**Outcome**	Outcomes are the changes in the context that result from how people respond to the intervention and the contextual mechanisms.
**Retrodiction**	Determining what mix of mechanisms interact, reinforce, moderate, or counteract to produce the outcomes.
**Retroduction**	Inferential thinking – what makes the observable phenomena possible, what must the real be like. Identifying the individual causal powers.
**Structure**	Material, physical, and human resources and their associated practices at the level of social and systems integration, that is, the relations between members of society and the relationship between institutions
**Transitive and Intransitive Dimensions**	The intransitive dimension is the physical and social world we inhabit; things are as they are and are not constituted by our understanding of them and are independent of how we describe them. The transitive is the theories and discourses we hold in order to understand our world.
