# Supplementary material for: A protocol for a critical realist synthesis of school mindfulness interventions designed to promote pupils’ mental wellbeing
Source: Front Public Health. 2024 Jan 9;11:1309649. doi: 10.3389/fpubh.2023.1309649 (PMC10803664; doi:10.3389/fpubh.2023.1309649)
Supplement: Supplementary file 4 [file Data_Sheet_4.PDF]

Supplementary Material 4:
Literature Review for Eliciting the Initial Programme Theory

Introduction
This paper discusses the literature that we drew on to develop the initial programme theory for a critical realist synthesis on how universal (health promotion) school-based mindfulness interventions[1] do (or do not) promote pupils' mental wellbeing. A SBMI is a social programme introduced into schools to solve a problem; the intervention mechanisms (mindfulness tools) can counteract the problem mechanisms, thereby making the desired changes happen or block them preventing any change. A critical realist programme theory is a middle-range theory (1) that seeks to understand how and why the intervention worked (or did not work) by identifying the underlying mechanisms triggered by actors' response to the intervention. It recognises that complex underlying mechanisms, structures, and contextual factors influence outcomes. The review focuses on universal interventions, SBMIs taught as a whole class or whole school intervention. The findings from the review will inform the programme theory for delivering a whole-school mindfulness intervention in primary schools in Ethiopia and Rwanda[2] (2,3). The literature includes empirical research reporting on evaluations of universal SBMIs and existing theories of mindfulness that may form part of the critical realist programme theory.

Our programme theory will be based on: (a) Margaret Acher's critical realism and the Structure, Agency, Context (SAC) Framework and (b) Context, Agency, Intervention, Mechanisms, Outcome configuration (CAIMO) (4). Central to Archer's theory is the recognition that agents make choices within structural and cultural constraints, i.e., actors make things happen (5). Individuals engage in a dialogue with themselves (an internal conversation) to make decisions, form opinions and understand their motives. They think about and critically evaluate their actions and circumstances in relation to the broader social context. Agents bring about change by triggering mechanisms that shift the underlying social structures, patterns of social relationships and culture.

Mindfulness interventions may give agents the tools to trigger individual, relational and social mechanisms thereby changing the structural and cultural context in which they live their lives. Mechanisms are *emergent*; that is, mechanisms may combine to form new mechanisms that are not reducible to the mechanisms from which they were formed. However, the contexts in which interventions are delivered vary, and mechanisms may not be triggered in any given context. Moreover, other mechanisms in a context may block the activation of new mechanisms. This requires the identification of theories that reach beyond mechanisms underlying the programme to obtain insights into the structural and cultural conditions that may influence the actors involved in a SBMI: pupils, teachers, school administrators, parents, and community members.
We operationalise Margaret Archer's theory using the intervention-context-actor-mechanisms analytic tool (Figure S24.1 & Table S4.1) (6,7). The tool links the intervention to context, agency, mechanisms, and outcomes (change in the person and the context). Although we use the tool for analytical purposes, it is not always easy to isolate and disentangle the effects of

---

[1] There have also been mindfulness interventions for teachers and training teachers in mindfulness and there is some evidence that training teachers can have a positive impact on pupils' wellbeing (97)
[2] Primary schools in Rwanda are intended for children from 7 to 13 years and in Ethiopia from 7 to 14 years. However, in both countries repetition and temporary dropouts means that a significant proportion of the pupils are older than the official completion age. In 2018 the net primary school enrolment rate was 85 and the gross 106 in Ethiopia and in Rwanda the net rate was 95 and the gross rate 141(132).

the intervention, the role of agency, the mechanisms of change and the context in which the change occurs. Multiple interacting factors are at play, change is often non-linear, and interventions can have unintended consequences, making it challenging to identify causality.

Figure S4.1: Intervention-context-actor-mechanisms (ICAM) Analytic Tool

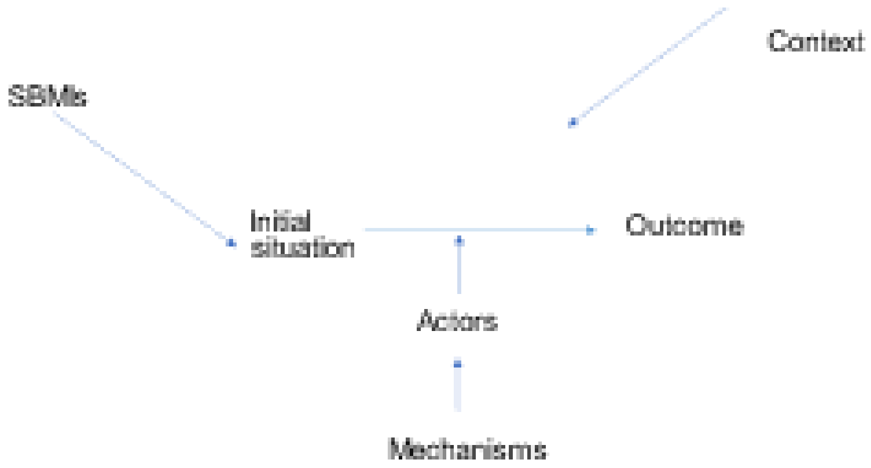

Table S4.1: Terms used in the intervention-context-actor-mechanisms analytic tool

Intervention	Programme elements and strategies designed to produce changes and promote pupils' mental wellbeing.
Context	The conditions (structural and cultural) that are likely to enable or constrain the activation of mechanisms. Contexts are dynamic, agenic, relational, immanent, historically located, and complex. As mechanisms are activated, the context changes. The change in context enables mental wellbeing outcomes from a SBMI to be sustained. Change may be non-linear and involve adaptation and feedback. Changes in context may occur due to factors other than the intervention.
Actors	The individuals, groups and institutions that play roles in the implementation and outcomes of interest and whose agency triggers the mechanisms that can change the context.
Mechanisms	Any underlying causal processes or social behaviour generated in specific contexts, including pre-existing mechanisms and ones triggered by actors' responses to the SBMI, that produce observable events or processes, e.g., a match lights when you strike it. Positive upward spirals (positive feedback loops) occur when mechanisms reinforce each other, leading to a cumulative positive outcome. Mechanisms can also counteract or inhibit new mechanisms (negative feedback loops) being triggered, blocking or limiting the impact of SBMIs. Mechanisms can be physical, social, psychological or a combination of these. Instead of just describing patterns or correlations, critical realists aim to identify the mechanisms that make the intervention work.
Outcomes	Outcomes are the immediate, intermediate, and long-term outcomes from the SBMIs that impact pupils' mental wellbeing in a given context. However, contexts are constantly subject to change through the interaction of structure and agency and the triggering of new mechanisms.

Literature Searches

To develop the initial programme theory, we systematically searched for systematic and scoping reviews of SBMIs (see Additional Material 1). We found only one systematic review that reviewed qualitative evaluations of SBMIs (8), although McKeering and Hwang, 2019) included an analysis of the findings from qualitative research in their systematic review of SBMI. We augmented the systematic searches with purposive searches, using Google Scholar and based on the Intervention-context-actor-mechanisms analytic tool. We searched for articles that could shed light on intervention mechanisms, the context for the interventions, the mechanisms triggered by the intervention or already in the context that influenced outcomes, and outcomes. We found no papers that reported a critical realist or realist evaluation of a SBMI. We also did purposive searching for theories that have been used to explain why mindfulness is expected to impact pupils' mental wellbeing.

The initial programme theory developed for our review of this literature will be tested and revised based on the findings from the critical realist synthesis.

Secular Mindfulness in Schools

Mindfulness is experiential and is both a process and an outcome. It is an inherent human capacity and a practice for enhancing this capacity (9,10). There is no substantive definition of mindfulness at the level of the individual or the organisation (11,12). However, mindfulness is generally described as the awareness that emerges when we intentionally pay attention to our experiences in the present moment with curiosity, acceptance and kindness (9,13). However, in mindfulness research and practice, there are diverse basic assumptions and theoretical traditions (14,15).

The empirical research, mainly conducted in adult populations, suggests that mindfulness has beneficial outcomes and promotes wellbeing (10). Furthermore, it is one of the few interventions shown to improve wellbeing. It has been shown that there is an association between the intervention and enhanced wellbeing (16). Wellbeing is a positive state, not just the absence of illness, and means feeling good and functioning well in one's daily life (17). Positive wellbeing (flourishing) is multidimensional and includes eudaemonic (positive functioning - seeking meaning, personal growth, and self-realisation) and hedonic (positive feelings - pursuing pleasurable experiences and positive emotions) aspects of well-being (17–19) and harmonic wellbeing (contentment, inner peace, harmony and balance (20). Elevated levels of wellbeing are associated with positive outcomes, including improved learning, productivity and creativity, good relations, prosocial behaviour and good health, and life expectancy.

The positive outcomes for adult populations led to mindfulness interventions being adapted for children and adolescents and recommendations that mindfulness should be taught in schools to promote child and adolescent wellbeing. Over the last twenty or so years, mindfulness interventions have been introduced in schools across the globe (21). Evaluating the impact of mindfulness interventions in schools suggests that, at least in some contexts, mindfulness taught in schools can promote pupils' mental wellbeing (see Additional Material 1).

However, it is necessary to recognise the difference between using mindfulness in clinical practice and whole-school mindfulness interventions. In clinical practice, the aim is to change the individual, to treat an individual with a problem, for example, depression, stress, or an

eating disorder, to alleviate their pain, if not cure them. The outcome of the intervention is the change in the individual's mental health that can be attributed to the mindfulness intervention. Whole school mindfulness interventions, however, aim to promote the mental wellbeing of all school community members. SBMIs are social interventions that aim to change the behaviour of the school community, transforming the school into a social institution. SBMIs are relational and embedded in an organisational and social context. Positive change in any individual depends not just on changes in other individuals but also on the structure of social relations and cultural values. Psychological theories that may be adequate to explain differences in individuals in clinical settings are not sufficient for explaining changes in the social context.

Types of School-based Mindfulness Interventions

SBMIs have been introduced into schools to foster pupil's and teachers' mental, behavioural, and emotional health, enhance concentration and academic performance, promote emotional regulation, and foster resilience (12,21). They are taught and practised in a secular or non-religious manner. The core concept of secular mindfulness is to focus on the present moment, cultivate awareness, and develop a non-judgmental attitude towards one's thoughts and experiences. However, no universally agreed-upon definition of a school-based mindfulness programme regarding content or delivery (15,22–25). Content and delivery are usually done through various activities, including psychoeducation, cognitive behavioural exercises, and experiential mindfulness practices such as guided mindfulness meditation, breathing exercises, body scan and hatha yoga. SBMIs explicitly aim to cultivate mindfulness in pupils and promote mental wellbeing by supporting learners to become more attentive (attention regulation), more empathetic and respectful of others, improve their interpersonal skills and resolve conflict peacefully, and better able to control their emotions, and to develop a more positive view of self and a more positive outlook on life (26,27).

SBMIs are generally what Ergas (2019b) refers to as mindfulness *in* education. They are designed to support the functioning of the school and the wellbeing of pupils and teachers and are often justified in economic terms. However, whole school and contemplative mindfulness interventions are generally closer to what Ergas refers to as mindfulness *as* education, aiming to create a more supportive environment that values the whole child's development (Table 2). A distinction can be made between mindfulness interventions that are more focused on transforming the individual and those that aim to transform the individual and the culture and structure of the school through whole-school interventions (15,28–31). From this perspective, schools collectively respond to the 'social problem' of child and adolescent mental wellbeing (32), challenging the criticism that mindfulness programmes individualise responsibility for wellbeing (33).

There are three main types of SBMIs: mindfulness-based stress reduction (MBSR), mindfulness-based cognitive therapy (MBCT) for children, and mindfulness-based social and emotional learning (MBSEL) (34). Some combine elements of more than one and/or combine with other social and emotional learning (SEL) interventions. MBSR and MBCT require facilitators to be trained and practice mindfulness, and pupils are expected to practice mindfulness techniques outside of formal lessons, but these are not requirements for MBSEL. One systematic review of SBMIs identified 36 different programmes (28). Thirty per cent (30%) of these were adaptations of MBDR or MBCT, 50% were novel programmes incorporating content from neuroscience, social and emotional learning, and positive psychology, and 20% involved brief practice without a formal programme. The extent to

which different outcomes are associated with varying mindfulness programmes is unclear from systematic reviews.

Mindfulness in education can be framed and implemented in diverse ways. SBMIs can be standalone programmes, or mindfulness can be embedded in the curriculum (35). SBMIs vary by the amount of training teachers have, if external experts or classroom teachers teach them, the content of the programme, the fidelity of intervention (the extent to which the teachers adhere to the intervention protocol), 'dose' (number and length of sessions), the amount of practice required, quality, reach (targeted, whole class[s], whole school, contemplative) (Table S4.2) (28,36–38). There is some evidence that the whole school approach provides a more transformative and sustainable model than the universal one (39).

SBMIs are then heterogeneous and include different curricula and programmes with no agreement on the core (essential) components, that is, *'the essential aspects of a programme including the practices, processes, and principles that are hypothesised to be causally linked to the measured outcomes' differ* (40). This lack of operational specificity in SBMIs is likely to be one reason the effects of interventions differ.

TableS4. 2: Types of School-based Mindfulness Interventions

**Approach**	**Description**
Targeted	The intervention is designed for small groups or individual pupils at risk of mental health problems or behavioural issues. The curriculum is usually customised to meet the needs of the pupils and taught by trained professionals with expertise in mindfulness and mental health.
Universal	Mindfulness practices are introduced to all children in a class, age group or the whole school regardless of their specific needs or characteristics. The curriculum is taught by trained classroom teachers or external facilitators during the regular school day. The length of the intervention can vary from a few hours to a course lasting up to 12 weeks. Mindfulness may also be integrated into the school's curriculum and everyday routines.
Whole School	Mindfulness practices and principles are integrated into the school's culture and practices by, for example, integrating it into the curriculum, into communication and discipline precesses, and the intervention aims to benefit pupils, teachers, and other school staff. There is universal participation in mindfulness practices with support from senior management, classroom teachers trained in mindfulness strategies and principles. Mindfulness is integrated into the curriculum, and mindfulness principles are applied to school discipline and conflict-resolution strategies. A whole-school mindfulness intervention aims to create a more mindful and supportive environment that fosters the well-being of the school community.
Mindfulness Contemplative Education	This whole school approach incorporates contemplative practices involving reflective and introspective activities that encourage deep thinking, self-inquiry, and mindfulness practices. The aim is to create a more mindful and compassionate learning environment that supports all school community members' personal and academic growth and recognises the importance of nurturing intellectual and emotional intelligence for a more rounded education.

(12,15,47–49,21,25,41–46).

Developing the Programme Theory

There is a lack of understanding of how SBMIs work and how pupils and their teachers exercise agency and produce outcomes (or not) (15). Experimental psychologists have dominated school-based mindfulness research, although it is recognised that mindfulness requires interdisciplinary/ transdisciplinary research, as does mental wellbeing research more generally (10,15,50,51). It is impossible to adequately research the impact of a social programme by only researching the possibility of changing the attitudes and behaviour of individuals. Investigating the potential to change context, social relations, norms, and values is also necessary.

Critical realism, which argues that access to reality can only occur through fallible theories, is not reconcilable with psychological positivism, which is prone to the ontic falacy (not recognising that our research is mediated by the limits of our knowledge and the influence of our culture and norms) and epistemic falacy (confusing statements about our ability or beliefs with statements about the objective reality of the world). It assumes it has access to an objective, neural and invariant reality external to the research process and based on the individual's biological and behavioural characteristics, which can be observed and studied. Nevertheless, to develop a critical realist programme theory, it is necessary to identify existing theories and theories of change that may form part of the critical realist programme theory and analyse the literature using the ICAM analytic tool to identify tentative CAMO configurations.

Following these four interrelated considerations inform the development of our programme theory. Firstly, the social world is complex and an open system. Secondly, we live in a laminated system of strata; that is, multiple levels of reality are essential to understanding why mindfulness interventions have the impact they do. Thirdly, mental wellbeing is influenced by biological, psychological, and social mechanisms – the biopsychosocial model of mental health. Fourthly, there is a need to develop interdisciplinary theories of how mindfulness works to promote mental wellbeing and enable individuals to flourish.

Social programmes are introduced into open or at least partially open systems, complex contexts where there is an interplay between the institution (social relations, norms and values existing before the programme is introduced and while it is being delivered) and the individual agency that can trigger new generative mechanisms (52,53). It is impossible to have closure, to control a social system and thereby test for invariant empirical regularities. It is impossible to isolate, e.g., schools from outside influences or known and calculate any such influences nor ensure consistency of inputs to different individuals, classes, or schools. Many biological, psychological and social mechanisms are concurrently active, with some mechanisms reinforcing and some frustrating others (54). There is the potential for existing mechanisms to block or partially block the triggering of new generative mechanisms or for generative mechanisms to be triggered and combine with each other and /or existing mechanisms to increase the impact of the intervention. This makes the outcomes of mindfulness interventions unpredictable in detail. However, there can be semi-regular-trends, demi-regularities (53); for some people in certain contexts, a mindfulness intervention will have the same impact on mental wellbeing.

Nature is stratified, and we live in a laminated system of strata (Figure 2) (55). Different and distinct layers make up both physical and social systems. The laminated system is formed by the emergent mechanisms generating multiple levels of analysis, with the whole being more than the sum of its parts (56). The system of interconnected mechanisms interacts within and

across each stratum. The relations between less and more basic strata are one-way relations of inclusion; all animals are composed of chemical substances, but not all chemical substances are parts of animals. Animals are not reducible to their chemical makeup, they can do all sorts of things which chemicals cannot do, but they cannot break the laws of chemistry. Anything belonging to a higher stratum is grounded by more than one kind of law; more than one mechanism is operating in the strata. At the level of actual relations, strata overlap and interact and affect each other in complex ways. Higher-level mechanisms are routed in and emergent from more basic ones; that is, they have properties which are not reducible to their lower-level parts. For example, team members can do things that individual members cannot. For critical realists emergence is relational; new mechanisms arise based on internal relationships (57).

Tikly (2015) has developed a laminated learning system in which each stratum is nested within another stratum, which we have adapted to take account of wellbeing (Figure S4.2). This takes account of the biological and psychological make up of each child and the social system, the latticework of roles and structures within which leaning takes place. Schools themselves are located in wider social, economic and political contexts that not only can have negative impacts on the mental wellbeing of (some) children and adolescents but also provide the broader context within which schools are located and influence how they operate (15,58,59).

FigureS4.2: A Laminated CA Learning and Wellbeing System

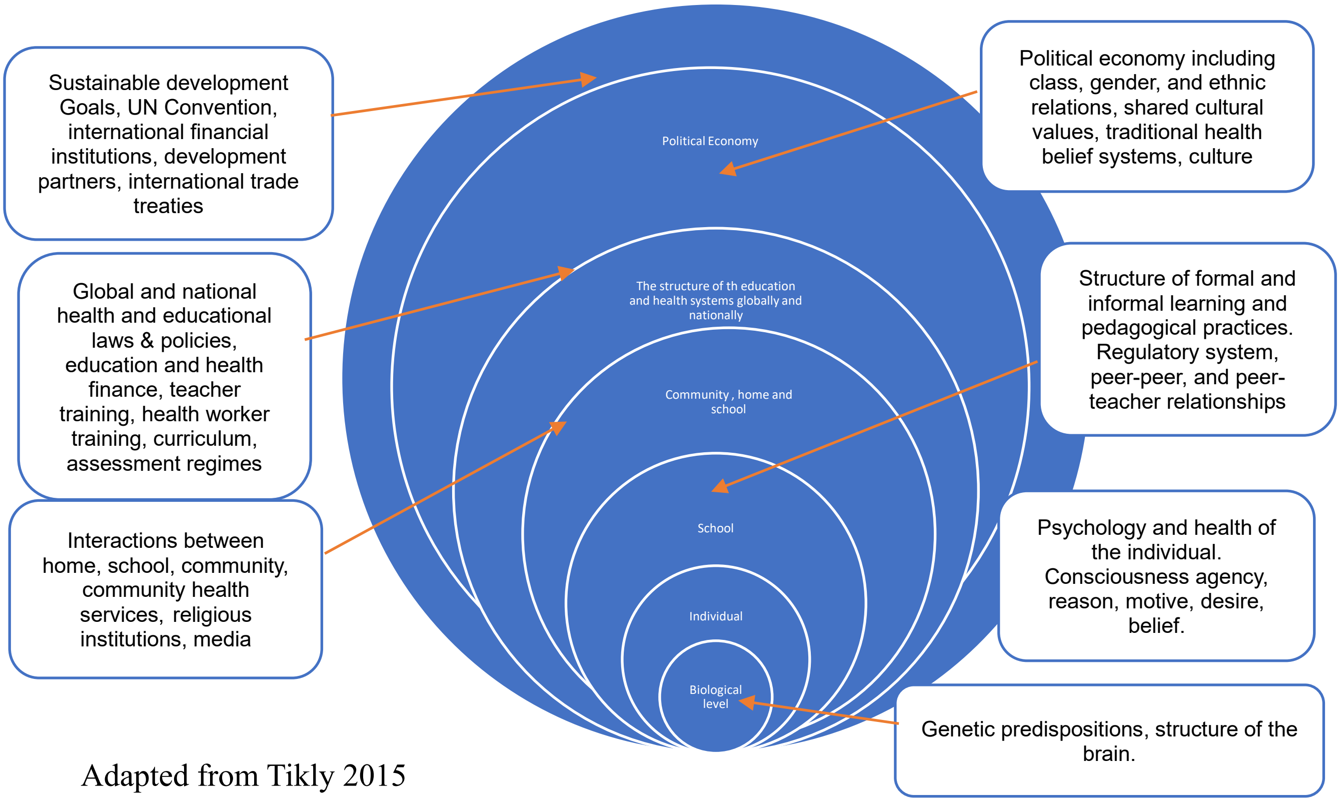

Adapted from Tikly 2015

Research on mindfulness in schools has mainly been from a psychological perspective, measuring changes in pupils as individuals rather than considering the impact that structural and cultural factors within the school, the community and the wider system may have on the effects of the intervention. However, limited attempts have been made to theorise relational

and ecological factors, including the structure and culture of schools and classrooms and the wider social context in which schools are embedded (15,60,61).

Taking a broader sociological lens enables us to see SBMIs as part of the Health Promoting Schools' (HPS) initiative driven by the World Health Organization (32,58,62). The HPS programme acknowledges the critical role of the school in promoting the physical and mental health of children and adolescents. The WHO laminated system for understanding an educational (school) health-promoting system includes government policies and practices, school policies and resources, school and community partnerships, and four elements of the school: the school curriculum, the school physical environment, the school health service, and the school social-emotional environment.

The biopsychosocial model of mental health is a holistic framework that recognises that health and wellbeing are influenced by biological, psychological, and social and epidemiological factors that interact and contribute to an individual's overall wellbeing. George Engle introduced it to avoid reductionism in health sciences (63). Biological factors include genetics, physiology, and physical health. Psychological factors encompass mental and emotional states, thoughts and behaviours, and social factors include cultural, socioeconomic, and environmental influences. The model acknowledges the complexity of the interacting processes that generate human health and the necessity of an interdisciplinary approach to understanding complex issues. However, it has been criticised for (1) underestimating the complexity of the process of integrating information from different systems, (2) not providing a guide to clinical practice, (3) not having a metatheoretical grounding (50) and (4) in practice retaining a strong residue of medical positivism (64)

While the model provides the potential for developing interdisciplinary theory in practice, this has not materialised partly because of the different views of causation held by researchers from different disciplines. The dominant 'black box 'approach in mindfulness research, as in metal health and wellbeing research more generally, mitigates against multidisciplinary research teams developing an interdisciplinary theory because there is no basis for integrating the disciplinary theories into a new interdisciplinary one. Understanding causation is essential if interventions are to promote mental wellbeing or aid recovery from poor mental wellbeing (50). The 'black box' approach argues that research should focus on input and output and that it is unnecessary to conjecture about how the intervention worked. However, a critical realist mechanisms approach argues that research should uncover the underlying mechanisms that produce the outputs and provide the basis for developing multidisciplinary theory using the laminated system (Figure 2) (50,55).

Critical realists argue that interdisciplinary research is undertaken by researchers from (through the lens of) all relevant levels in the laminated system needed to answer the research questions (50). However, the biological, psychological and social sciences have distinct subject matter and types of generative mechanisms which cannot be reduced to one another (55). Therefore, interdisciplinary research must analyse structures, mechanisms, and outcomes at each level (biological, psychological, and social) using the study design and methodology appropriate for the respective levels (65). The challenge is to integrate knowledge about the interplay between structures and mechanisms from different levels to provide a broader understanding of a complex phenomenon, avoiding reductionism, atomism and holism (50,54,59).

Theories of Mindfulness

Roeser *et al.* discuss two meta-theories underlying mindfulness research in education (15). The dominant meta-theory is Cartesian, based on the philosophical principles of Descartes, which tends towards dualism (mind/body split), reductionism, and a mechanistic view of the world. In mindfulness research, this means investigating how mindfulness practices affect mental processes separately from physical wellbeing, adopting a sceptical approach and methodological rigour, and examining mindfulness in reductionist terms as a cognitive process. Post-Cartesian meta-theories, by contrast, emphasise holism, contextualism and culture, subjective experience, complexity and emergence, interdisciplinary collaboration, and ethical and social implications. From this stance, mindfulness is a *social practice* learned socially through interaction with others (15).

Psychologists have drawn on several perspectives to guide mindfulness research and interpret findings. However, the theoretical justification for mindfulness interventions to promote mental wellbeing in schools and other nonclinical settings remains limited (66,67). Research on SBMIs has focused on researching if interventions work rather than theorising why and how they have impact (11,38,68–72). They are generally concerned with asking if they work with mindfulness programmes (interventions) regarded as a 'treatment' that will promote/improve pupil (and teacher) mental wellbeing. The reason most frequently given for hypothesising that a SBMI will promote pupil mental wellbeing is that mindfulness has been proven to positively impact adults (Zoogman et al., 2015) positively. More generally, SBMIs are seen as a positive education intervention (73) broadly congruent with the principles of positive psychology (39,74) and with the social and emotional learning framework (75,76). Positive psychology studies positive emotions and other factors contributing to human wellbeing, including resilience, positive relationships, personal growth, engagement in activities, and meaning and purpose in life. It aims to provide practical tools and interventions to help individuals and communities lead happier and more fulfilling lives. The social and emotional learning framework is designed to enable pupils to develop social and emotional skills to promote their wellbeing and enable them to develop to their full potential. The core components include self-awareness, self-management, social awareness, relationship skills, and responsible decision making. It is integrated into the school curriculum, taught by teachers in a safe and supportive learning environment, and, in the case of whole school interventions, involves the whole school community.

Using a targeted search strategy, we found a few papers that referred to a theoretical perspective that guided research on SBMIs or, more generally, children and adolescents. Shute (2019), for example, argues that mindfulness programmes have been introduced into schools without any psychological development perspective on the practice. The cognitive development theories of Piaget were used in two papers (25,77) and Vygotsky in three (25,77,78). Others include Maslow's hierarchy of needs (76,78), Roger's humanistic perspective (76,78) and broaden-and-build theory (79,80). The theories are not seen as mutually exclusive. Shute's (2018) theoretical framework links a model of mindfulness as metacognition with Piagetian, Vygotskian, and dynamic systems theories of development. Griffin's (2022) theoretical framework draws on Vygotsky's sociocultural theory of learning, Maslow's hierarchy of needs, and Rogers' emphasis on the necessary attributes of an environment that facilitates the growth of an individual. Kazanjian (2022) uses a multicultural humanistic psychological theoretical framework drawing on humanistic psychologists including Rogers, Moustakas, Buhler and Maslow as well as multicultural scholars.

Psychological theories have provided non-mutually exclusive theories of how mindfulness practices work in clinical and nonclinical adult populations. We have identified nine and the realist theory developed by Micklitz et al. (2021) for work-based stress reduction interventions (Table S2.3). Reperceiving/decentring, the nonreactive, observation of thoughts, emotions and sensations as passing events is considered the central change mechanism in mindfulness (10,13,81,82). It provides a space between stimulus and response, reducing automatic reactivity and enabling an area in which choices can be made and acted on. Knowing that choices can be made and acted on will likely increase a person's sense of autonomy and self-efficacy. As a meta-mechanism, it is thought to activate related mechanisms of self-regulation (attention, emotion, and behaviour), experiential exposure, and values clarification. Madonna (2018), in her qualitative study with past participants of a MBSR course, found that respondents described using reperceiving, that is, becoming more aware of their initial intentions and using this awareness to respond in new ways, describing meta-cognitive awareness of the quality and validity of their thoughts, and describing the quality of their attitudes toward their own experiences.

.

TableS4.3: Psychological Theories Used in Mindfulness Research with Adults and A Realist Programme Theory of Stress Reduction in the Workplace

Attention Regulation Theory	Mindfulness works by helping people to become more attentive and thereby more aware of their thoughts, emotions and physical sensations without judgement (83).
Cognitive Behavioural Theory	Mindfulness enables awareness and development of a non-judgemental attitude to automatic thought patterns, breaking the rumination cycle and promoting mental wellbeing (84).
Default Mode Network Theory	Neuroimaging research has shown that mindfulness can alter the default mode network (network of interconnected brain regions) associated with referential thinking and mind wandering, leading to decreased rumination and improved attention (85).
Emotional Regulation Theory	Mindfulness reduces emotional reactivity and improves emotional wellbeing by increasing awareness of emotions as they arise and observing them without judgement (86).
Monitor and Acceptance Theory	Mindfulness enhances awareness of experiences and acceptance, and these skills together explain how mindfulness improves negative affectivity (experiencing negative emotions and moods), stress and stress-related health outcomes (87)
Neuroplasticity Theory	Neuroplasticity is the brain's capacity to change in response to learning, experience, and environmental influences throughout life. Regular mindfulness practice has been linked to changes in the brain structures associated with memory, learning, and emotional regulation (88).
Resilience Theory	Mindfulness enhances the ability to recuperate from and withstand hardships by mending oneself (89).
Reperceiving	A cognitive process that involves seeing something from a new or different perspective. Changing one's perception of a situation, thought or emotion by observing it from a more objective or detached position. Mindfulness practice can develop people's skills in reperceiving (13).
Self-determination Theory	Mindfulness can enhance intrinsic motivation and wellbeing by satisfying basic psychological needs for autonomy, competence and relatedness (90). Mindfulness confers a range of intra-and

	interindividual benefits, including wellbeing and pro-sociality, in part due to the positive link between mindfulness and autonomous motivations (people finding more interest or value in aspects of their lives) (67,91).
Stress Reduction Theory	Mindfulness practices reduce stress's psychological and physiological impact by promoting relaxation and changing the relationship to stressors (92).
Realist Programme Theory of Workplace Mindfulness-Based Programmes	'Workplace MBIs help individuals build resources to better deal with stress/distress and enhance wellbeing. Participants (and management) must invest resources (e.g., time, money, and energy) to build these resources. Moreover, by investing in an MBI at work, employees might put existing resources at risk, such as completing work tasks, image of strength and perfection, or fitting with the team. Management must, therefore, weigh the benefits of developing new resources against the threat of potential resource loss. In line with the conservation of resources (COR) theory, individuals seem to be more likely to invest in a workplace MBI if: (1) the programme is seen to help them attain goals; (2) mindfulness complements existing resources and (3) the environment is supportive of their engagement with the programme. For an environment to be perceived as supportive, it is essential that employees feel safe. At each stage of an MBI, psychological safety functions as a 'door opener' for the subsequent, extended, or deepened engagement with the intervention. 'Feeling safe' in the group or about the MBI instructor might be necessary to develop acceptance/compassion and, subsequently, to bring mindfulness to challenging work situations. Applying COR theory to workplace MBIs suggests that if the environment is not supportive, employees might prefer to protect current resources (i.e., time for work, status, free time) instead of investing in developing new ones (i.e., mindfulness). They might use mindfulness strategically (e.g., combining brief exercises with other routines or breathing techniques for coping in stressful situations), which may benefit them but not as much as with deeper engagement. If, on the other hand, a MBI is seen to fit with an individual's or organisation's goals, if it complements existing resources and if the setting is perceived as safe, a workplace MBP might set off gain spirals with a positive impact on a wide range of well-being outcomes (93).

Mindfulness Interventions in Schools - Theories of Change

Research on SBMIs has mainly looked at outcomes, and there has been little process evaluation, qualitative or quantitative (38,68–70). Although most impact evaluations of SBMIs have been empiricist, measuring cause and effect, some have opened the 'black box' using theories of change (Table S2.45). They still reduce reality to empirical observations; they apprehend and define the real as identical to empirically grounded research and assume cause and effect but identify moderators (including contextual and intervention factors), mediators, and mechanisms. While this remains thin evidence, it can provide a starting point for retrodiction and retroduction for asking how agency, in response to the intervention,

brought about changes. Our targeted searches identified three theories of change for SBMIs (15,38,72,94,95) and a systematic review of moderators and mediators in SBMIs (Tudor *et al.* 2022). Other researchers have hypothesised that the outcome of SBMIs, the pathway to improved mental wellbeing, is mediated by a reduction in involuntary stress responses/improved emotional regulation (96). Researchers have also stressed that pupils' mental wellbeing can be promoted when teachers are trained in mindfulness as well as or independently of pupils (36,72,97,98). Lavy and Berkovich-Ohana (2020) argue that education research shows that sustainable and scalable changes in pupils are almost always related to changes in the environment and, more specifically, to changes in their teachers' mental wellbeing. Promoting teachers' mental wellbeing enables them to use mindfulness tools to trigger mechanisms that generally change the structure (relationships) and culture of the classroom and the school.

TableS4.4: Terms Used in Mindfulness Theories of Change

Intervention	Programme elements and strategies designed to produce changes and promote pupils' mental wellbeing.
Theory of Change	A structured framework is used to plan, implement, and evaluate interventions. It outlines the expected logical sequence of inputs, activities, outputs, outcomes, and anticipated impacts to lead to the desired change. It focuses on the linear cause-and-effect relationships, not the underlying causal mechanisms.
Logit Model	A diagrammatic representation of the theory of change.
Mediator	A mediator is a variable or process that helps explain the relationship between two other variables. It helps to understand the pathway/causal chain by which the intervention affects the outcomes. Mediation analysis investigates the intermediate steps or mechanisms between the intervention and the outcomes, the *pathway* between cause and effect.
Mechanisms[1]	A mechanism is the underlying process, system or functions explaining why the mindfulness intervention works. Mechanisms are often theoretical constructs used to describe the inner workings or functions of psychological processes that produce the outcomes from the mindfulness intervention. The processes are studied to understand how the intervention works. Mechanisms are based on observable data and research findings[2], focused on explaining specific psychological processes and used to develop testable hypotheses and models.
Moderator	A moderator is a variable that researchers suspect may influence how the treatment affects the outcome. It could be a demographic characteristic (e.g., age, gender), a contextual factor (e.g., location, time of intervention), or any other relevant factor.

Notes: [1]The use of 'mechanism 'in psychology differs from that in critical realism. In psychology, mechanisms explain specific psychological processes and behaviours. Critical realists use (generative) mechanisms as ontological concepts to describe the underlying hidden causal powers that make things happen in the world.
[2]In empiricism, the measurement of any behaviour or experience that can be conceptualised and operationalised by self-reports on inner states is accepted as empirical evidence (99).

Andreu and Garcia-Rubio (2019) have developed a theory of change, a logit model predicting the pathway through which SBMIs work, the *Integrative Model of Outcomes and Mechanisms of Change in the MBIs in the Classroom*. They propose that SBMIs directly affect specific processes that mediate intervention-distal effects. They identify three types of

mediators. Firstly, mindfulness skills, mental and body awareness, acceptance, and psychological flexibility. Secondly, cognitive and emotional processes, attention, executive functions, and self-regulation. Thirdly, social competencies, prosocial behaviours and dispositions, self-kindness and compassion, empathy, gratitude, altruism, reduced behavioural problems, aggression, and anger. They also propose that mindfulness interventions are moderated by 'dose' (number and length of sessions), amount of practice, acceptability of the intervention, quality of the intervention, age and gender of the pupils and baseline levels of mindfulness. The outcomes they predict are mental health (wellbeing, self-concept), peer relations (peer acceptance, peer support, reduction in bullying), student engagement (improved classroom behaviour, improved classroom climate, readiness to learn), and improved academic performance (Andreu and Garcia-Rubio, 2019).

They have tested the model specifically focusing on pupils' socio-emotional and academic development in a universal cluster-randomised control trial involving 313 elementary school pupils (7 to 12 years) in two schools in Madrid (Spain) using the GrowUP Breathing programme (95). The findings aligned with the Integrative Model of Outcomes and Mechanisms of Change predictions *in the MBIs in the Classroom.* They found that the children in the intervention classrooms improved their mindfulness, socio-emotional, wellbeing, and academic skills. They also found that the SBMI showed indirect effects through emotional regulation on pupils' emotional symptoms, provinciality, peer relationship problems, emotional engagement, and behavioural engagement.

Tudor *et al.* (2022) developed a comparable conceptual model (pathway of change) of moderators, mediators and implementation factors based on a systematic review of universal SBMIs. However, the findings were limited by only five of the papers they reviewed evaluating potential mediators, and most of these did not study changes in the mediator before testing for the change in the outcome. Their model is comparable to Andreu's and Garcia-Rubio's (2019). However, they recognise that the wider context in which the school is located and the characteristics of schools are important moderators of outcomes. They also recognise that school and classroom climate changes may mediate programme effectiveness, including improving teachers' mental health. In terms of mediation, their analysis suggested mindfulness and cognitive reactivity might mediate the effect of SBMIs on mental health, healthy relationships, and performance.

Lavy and Berkovitich-Ohana (2020) stress the importance of changing the school structure and culture for mindfulness interventions to be sustainable. They propose a theory of change, a causal chain from practising mindfulness to increased teacher wellbeing and effectiveness and increased pupil wellbeing and social and academic development. They suggest that practising mindfulness decreases self-centeredness, followed by increased caring capacities, emotional regulation, empathy, and compassion, improving teacher-pupil relationships and leading to positive outcomes.

From a contemplative education perspective, Roeser and colleagues (11,15,94) argue that it is necessary to go beyond universal SBMIs and develop *mindful schools*. This standpoint asserts the goal is to transform not just pupils (promote their wellbeing and learning capabilities) but also the contexts in which children learn, the structure (social relations) and the school's culture. The school's transformation *sustains* and *automatises* the benefits of mindfulness education and practice, and the transformation of pupils is essential for supporting mindful schools. In their theory of change, the school leadership, teachers, and pupils are all trained in mindfulness. This enables upward and downward linking of mindful

leadership (vision and instruction) to develop a mindful school culture through teachers' mindfulness, a mindful classroom culture, and pupils' mindfulness. In a recent randomised controlled trial (RCT), this group found that educating teachers in mindfulness improves the classroom culture and pupils' wellbeing (97,100).

In addition, post-Cartesian psychologists see contemplative education as a transdisciplinary theory that explains the effects of engagement with mindfulness practices on the mind, brain, body, behaviour and social relationships across the lifespan It is a development perspective that aims to: (1) describe the effects of engagement on the body, brain, mind, and social relationships, (2) explain the effsects of mindfulness at the physical, psychological, and behavioural levels, and (3) use the descriptive and explanatory findings to optimise human development. From this perspective, mindfulness training enables learners to gain mindfulness skills, and once they have learned these skills, they can practice them in their social life. This enables the development of *mindful schools* where the culture is one of mutual support, cooperation, mindful, and allowing all pupils to achieve their potential (94). Research on universal mindfulness programmes in schools suggests that it is possible to create this culture and that it is this culture that promotes pupils' (and teachers') wellbeing (29,101).

While recognising the importance of the school environment, these theories of change still focus on individual-level psychological change rather than on how the SBMI changes the school environment. This is because they have not examined what generative mechanisms underlie the effects. They are insufficiently complex (102). Sustainability requires changing the context (the structure and culture of the school), which conditions pupils' (and teachers' and school administrators') behaviour. There need to be changes in ecological and individual-level processes to promote pupils' (and teachers') mental wellbeing.
Furthermore, theories need to explain how SBMIs give pupils' (and their teachers and school administrators) the tools to trigger the necessary changes. Bonell et al. argue that interventions to modify the school environment and improve pupils' health and wellbeing must integrate four pathways through which the school environment influences student wellbeing. These pathways are (1) student-school commitment (attachment), (2) student -peer commitment (pro- or anti-school peer attachment), (3) student cognition (practical reasoning, norms and attitudes, pro-anti-school, legitimacy of authority, pro- health or pro-risk health behaviours) and (4) student behaviours (pro-school/healthy behaviours and anti-school/risk behaviours. We need to develop a programme theory which explains how the changes Roeser and his colleges and Lavy and Berkovitich-Ohan envisage are brought about by pupils, teachers and school administrators using mindfulness tools to change the school environment.

Contexts of Action

In what circumstances do mindfulness interventions enable pupils (and their teachers) to trigger mechanisms that promote pupils' (and teachers') mental wellbeing, changing the school structure and culture, and the salient conditions likely to enable or constrain the activation of SBMI mechanisms? It comprises both the physical and the social environment that favours or does not favour the SBMI having the expected outcomes.
The contexts of schools are embedded in, and the structure and culture of schools and classrooms within these vary widely. The context is dynamic and relational, and a SBMI is introduced in a culture, place, and time (10). The immediate context for universal SBMIs is the classroom, but classrooms and schools are embedded in a wider context that can impact the delivery of SBMIs (see Figure 2 above)(15).

The review identified that, in general, little information is given about the wider contexts that SBMIs are introduced apart from references to the concerns about the mental wellbeing of children and adolescents. However, there is reference to the multilayered factors that may impact on implementation and effectiveness of SBMIs (35,37,103,104), including political support (105), community and family support (41,43) and funding, allocating time in the school timetable, staff buy-in, and relative prioritisation (106,107) as important elements of context for successfully introducing SBMIs. A positive school context with school leaders supporting SBMIs and with mindfulness integrated into a school culture prompting wellbeing, positive education and a longer-term strategic approach is also seen as necessary (35,46,107). The characteristics of students, class, gender, age, race and ethnicity and mental health status of the students are also identified as moderators (36).

Agency and Mechanisms

The literature points to the importance of the buy-in and commitment of school administrators and classroom teachers delivering SBMIs (25,35,108–110) and the quality of delivery (28,36). The response of students and the extent to which the programme engages and stimulates them so that they actively engage with it (36,106,108,110–112). Mindfulness can equip learners with agency and a sense of purpose and build competencies for contributing to their own lives and others (39). However, poorly trained, uncommitted teachers and lack of pupil engagement can be a barrier to pupils using mindfulness tools and triggering mechanisms that promote their wellbeing.

SBMIs explicitly aim to cultivate mindfulness in pupils and promote mental wellbeing by supporting learners to become more attentive (attention regulation), more empathetic and respectful of others, better able to control their emotions, and to develop a more positive view of self and a more positive outlook on life with the aim of promoting mental wellbeing (24,26,27,38,113).

The literature refers to psychological mechanisms hypothesised to bring about these changes. The most frequently mentioned moderators are self-regulation skills (executive functioning and emotional regulation) (28,44,95,108,114–117). Other mechanisms reported in the literature include cognitive reactivity (44,118), self-coldness (118), self- compassion (119), resilience (43,120), dispositional mindfulness (36,95,121,122), prosocial behaviour (44), self-efficacy (104), (a reduction in) rumination (123,124), stress reduction (31,44,125) and autonomy (117).

However, it is necessary to move beyond events and changes that can be empirically observed to uncover the generative mechanisms that cannot be directly observed to understand how and why change occurred. We can only do this by developing theories of how generative mechanisms are triggered by pupils and teachers that lead to the changes. Agency is the actions or practices of the pupils' teachers' school administrators, other individuals, groups, classes, or schools in response to a SBMI, how they use (or are thawed in using) the mindfulness tools in their everyday lives to trigger generative mechanisms. It is agents that make things happen, not interventions.

The starting point for theorising how the mindfulness intervention triggered generative mechanisms that promote mental wellbeing is identifying statements by actors explaining or justifying why they used a resource to achieve an expected outcome. The findings from qualitative research show how pupils have used mindfulness skills to trigger mechanisms that enable them to pay more attention (take more notice and reduce mind wandering), control

behaviour, be less forgetful, manage emotions, be more aware of the perspectives of others and respond rather than react to situations, and to relax (8,30,108,109,126,127). These skills enable them to build healthy relationships with fellow pupils and teachers, changing the structure and relationships in the classroom and school more generally (128). Teachers and school administrators report that they have been able to use skills gained through mindfulness to improve relations with others, remain calm in stressful situations and be more tuned into the needs of students. They report that students are more relaxed and focused and can focus more on the processes of student learning rather than on content and outcomes (129).

Outcomes

The psychological mechanisms that pupils trigger using the intervention tools cause observable changes in pupils (and their teachers), which have been identified in trials, including improved internal regulation, being less stressed, having better social and interpersonal skills, greater self-esteem and self-accept (36,130). Improvements in pupils' mental wellbeing, academic performance and behaviour have been identified as the primary outcomes. However, little consideration has been given to how the mechanisms may interact and combine, resulting in a spiralling of benefits.

Less attention has been given to the social benefits of mindfulness interventions and how the psychological mechanisms interact and combine to bring about social benefits, how the agency of pupils (and teachers) changes the context by improving the classroom/school environment, structure and culture (45,114,121,131). Pupils feel safer; there are more positive relations between pupils and between pupils and teachers are more positive, and there is a sense of community (43,44,47,108). The change in structure (social relationships) and culture enables pupils and their teachers to benefit from mindfulness interventions and improvements to be sustained (28,43).

References

1. Merton RK. "On Sociological Theories of the Middle Range.," *Social Theory and Social Structure*. New York, NY: Simon& Schuster, The Free Press (1949) http://www.csun.edu/~snk1966/Robert K Merton - On Sociological Theories of the Middle Range.pdf
2. Abbott P, D'Ambruoso L, Yared M, McNamee P, Hailu T, Nzabalirwa W. A Critical Realist informed pilot cluster control trial evaluating the effectiveness of a mindfulness intervention for promoting child and adolescent mental wellbeing in Rwanda and Ethiopia. *Res Regist* (2023) 8799: https://www.researchregistry.com/browse-the-registry#home/?view_2_search=8799&view_2_page=1
3. Abbott P, D'Ambruoso L, Yared M, McNamee P, Nzabalirwa W. Study protocol for a Critical Realist pilot cluster-randomised controlled trial of a whole-school-based mindfulness intervention (SBMI) promoting child and adolescent mental wellbeing in Rwanda and Ethiopia. *medRxiv* (2023) May:
4. Archer M. *Realist Social Theory: The Morphogenetic Approach*. Cambridge: Cambridge University Press (2008).
5. Archer M. *Structure, Agency and the Internal Conversation*. Cambridge: Cambridge University Press (2014). https://www.cambridge.org/core/books/structure-agency-and-the-internal-conversation/4E4164D9D8952F6163AD29CB86A23BF3
6. Mukumbang FC, Marchal B, Van Belle S, van Wyk B. Using the realist interview approach to maintain theoretical awareness in realist studies. *Qual Res* (2020) 20:485–515. doi: 10.1177/1468794119881985
7. Van Belle S, Abejirinde I-O, Ssennyonjo A, Srinivas PN, Hebbar P, Marchal B. How

to develop a realist programme theory using Margaret Archer's structure–agency–culture framework: The case of adolescent accountability for sexual and reproductive health in urban resource-constrained settings. *Evaluation* (2023) doi: 10.1177/13563890231185167

8. Sapthiang S, Van Gordon W, Shonin E. Health School-based Mindfulness Interventions for Improving Mental Health: A Systematic Review and Thematic Synthesis of Qualitative Studies. *J Child Fam Stud* (2019) 28:2650–2658. doi: 10.1007/s10826-019-01482-w
9. Kabat-Zinn J. Mindfulness-based interventions in context: Past, present, and future. *Clin Psychol Sci Pract* (2003) 10:144–156. doi: 10.1093/clipsy/bpg016
10. Cooper CL. "An Exploration of the Effects of Mindfulness Training and Practice in Association with Enhanced Wellbeing for Children and Adolescents.," In: Huppert FA, Cooper C, editors. *Wellbeing: A Complete Reference Guide, Volume 6 Interventions and Policies to Enhance Wellbeing*. Hoboken, N J: Wiley (2014) https://www.wiley.com/en-ie/Wellbeing:+A+Complete+Reference+Guide,+Volume+VI,+Interventions+and+Policies+to+Enhance+Wellbeing-p-9781118608357
11. Roeser RW. "Processes of Teaching, Learning, and Transfer in Mindfulness-Based Interventions (MBIs) for Teachers: A Contemplative Educational Perspective.," In: Schonert-Reichl KA, Roeser RW, editors. *Handbook of Mindfulness in Education Integrating Theory and Research into Practice*. Cham: Springer (2016) doi: 10.1007/978-1-4939-3506-2
12. Ergas O. Mindfulness In, As and Of Education: Three Roles of Mindfulness in Education. *J Philos Educ* (2019) 53:340–358. doi: 10.1111/1467-9752.12349
13. Shapiro SL, Carlson LE, Astin JA, Freedman B. Mechanisms of Mindfulness. *J Clin Psychol* (2006) 62:373–386. doi: 10.1002/jclp.20237
14. Daniel C, Walsh I, Mesmer-Magnus J. Mindfulness: Unpacking its three shades and illuminating integrative ways to understand the construct. *Int J Manag Rev* (2022) 24:654–683. doi: 10.1111/ijmr.12296
15. Roeser RW, Greenberg MT, Frazier T, Galla BM, Semenov AD, Warren MT. Beyond All Splits: Envisioning the Next Generation of Science on Mindfulness and Compassion in Schools for Students. *Mindfulness (N Y)* (2022) doi: 10.1007/s12671-022-02017-z
16. Lyubomksky S, Sheldon KM, Schkade D. Pursuing happiness: The architecture of sustainable change. *Rev Gen Psychol* (2005) 9:111–131. doi: 10.1037/1089-2680.9.2.111
17. Huppert FA, So TTC. Flourishing Across Europe: Application of a New Conceptual Framework for Defining Well-Being. *Soc Indic Res* (2013) 110:837–861. doi: 10.1007/s11205-011-9966-7
18. Diener E, Wirtz D, Tov W, Kim-Prieto C, Choi D won, Oishi S, Biswas-Diener R. New well-being measures: Short scales to assess flourishing and positive and negative feelings. *Soc Indic Res* (2010) 97:143–156. doi: 10.1007/s11205-009-9493-y
19. Ruggeri K, Garcia-Garzon E, Maguire Á, Matz S, Huppert F. Well-being is more than happiness and life satisfaction: A multidimensional analysis of 21 countries. *Health Qual Life Outcomes* (2020)1–16. https://hqlo.biomedcentral.com/track/pdf/10.1186/s12955-020-01423-y.pdf
20. Gallup and the Wellbeing for, Planet Earth Foundation. Wellbeing for All: Incorporating Harmonic Principles of Wellbeing in Subjective Wellbeing Research and Policymaking. (2023) https://www.gallup.com/analytics/510770/inclusive-wellbeing-research.aspx

21. Ergas O, Hadar LL. Mindfulness in and as education: A map of a developing academic discourse from 2002 to 2017. *Rev Educ* (2019) 7:757–797. doi: 10.1002/REV3.3169
22. Van Dam NT, van Vugt MK, Vago DR, Schmalzl L, Saron CD, Olendzki A, Meissner T, Lazar SW, Kerr CE, Gorchov J, et al. Mind the Hype: A Critical Evaluation and Prescriptive Agenda for Research on Mindfulness and Meditation. *Perspect Psychol Sci* (2018) 13:36–61. doi: 10.1177/1745691617709589
23. Crane RS, Brewer J, Feldman C, Kabat-Zinn J, Santorelli S, Williams JMG, Kuyken W. What defines mindfulness-based programs? The warp and the weft. *Psychol Med* (2017) 47:990–999. doi: 10.1017/S0033291716003317
24. Rempel KD. Mindfulness for Children and Youth: Review of the Literature with an Argument for School-Based Implementation. *Can J Couns Psychother* (2012) 46:201–220. https://mindfulnessinschools.org/wp-content/uploads/2013/09/remple.pdf
25. Moreno AJ. A Theoretically and Ethically Grounded Approach to Mindfulness Practices in the Primary Grades. *Child Educ* (2017) 93:100–108. doi: 10.1080/00094056.2017.1300487
26. Hölzel BK, Lazar SW, Gard T, Schuman-Olivier Z, Vago DR, Ott U. How does mindfulness meditation work? Proposing mechanisms of action from a conceptual and neural perspective. *Perspect Psychol Sci* (2011) 6:537–559. doi: 10.1177/1745691611419671
27. Shapiro SL, Lyons KE, Miller RC, Butler B, Vieten C, Zelazo PD. Contemplation in the Classroom: a New Direction for Improving Childhood Education. *Educ Psychol Rev* (2015) 27:1–30. doi: 10.1007/s10648-014-9265-3
28. Roeser RW, Galla BM, Baelen RN. Mindfulness in Schools: Evidence on the Impacts of School-Based Mindfulness Programs on Student Outcomes in P–12 Educational Settings. A Policy Brief for Robert Wood Johnston Foundation. (2022) https://prevention.psu.edu/wp-content/uploads/2022/09/PSU-Mindfullness-Brief-2022.pdf
29. Jennings PA, Frank JL, Snowberg KE, Coccia MA, Greenberg MT. Improving classroom learning environments by cultivating awareness and resilience in education (CARE): Results of a randomized controlled trial. *Sch Psychol Q* (2013) 28:374–390. doi: 10.1037/spq0000035
30. Luong MT, Gouda S, Bauer J, Schmidt S. Exploring Mindfulness Benefits for Students and Teachers in Three German High Schools. *Mindfulness (N Y)* (2019) 10:2682–2702. doi: 10.1007/s12671-019-01231-6
31. Gouda S, Luong MT, Schmidt S, Bauer J. Students and teachers benefit from mindfulness-based stress reduction in a school-embedded pilot study. *Front Psychol* (2016) 7:1–18. doi: 10.3389/fpsyg.2016.00590
32. WHO. Helping Adolescents Thrive: Guidelines on Mental Health Promotion and Prevention Interventions for Adolescents. (2020)
33. Nehring D, Frawley A. Mindfulness and the 'psychological imagination.' *Sociol Heal Illn* (2020) 42:1184–1201. doi: 10.1111/1467-9566.13093
34. Hosan NE, Smith V, Strean WB, Sibinga EMS, Punja S, Vohra S. The "what," "why," and "when" of using mindfulness in schools: Best practices and guidance for educators and policymakers. *Theory Pract* (2022) 61:465–476. doi: 10.1080/00405841.2022.2107822
35. Nguyen D, Kleeman NJ, Yager Z, Parker AG, Shean MB, Jefferies W, Wilson-Evered E, Pucinischi CP, Pascoe MC. Identifying barriers and facilitators to implementing mindfulness-based programmes into schools: A mixed methods study. *Appl Psychol Heal Well-Being* (2022) 14:1172–1188. doi: 10.1111/aphw.12329
36. Tudor K, Maloney S, Raja A, Baer R, Blakemore SJ, Byford S, Crane C, Dalgleish T,

De Wilde K, Ford T, et al. Universal Mindfulness Training in Schools for Adolescents: a Scoping Review and Conceptual Model of Moderators, Mediators, and Implementation Factors. *Prev Sci* (2022) 23:934–953. doi: 10.1007/s11121-022-01361-9

37. Emerson LM, de Diaz NN, Sherwood A, Waters A, Farrell L. Mindfulness interventions in schools: Integrity and feasibility of implementation. *Int J Behav Dev* (2020) 44:62–75. doi: 10.1177/0165025419866906
38. Andreu CI, García-Rubio C. "How does mindfulness work in schools? An integrative model of the outcomes and mechanisms of change of mindfulness-based interventions in the classroom.," In: Steinebach C, Langer AI, editors. *Enhancing Resilience in Youth*. Cham: Springer (2019). p. 139–157 doi: 10.1007/978-3-030-25513-8
39. Sheinman N, Russo-Netzer P. "Mindfulness in Education: Insights Towards an Integrative Paradigm.," *The Palgrave Handbook of Positive Education*. Cham: Springer (2021) doi: 10.1007/978-3-030-64537-3
40. Felver JC, Cary EL, Helminen EC, Schutt MKA, Gould LF, Greenberg MT, Roeser RW, Baelen RN, Schussler DL. Identifying Core Program Components of Mindfulness-Based Programming for Youth: Delphi Approach Consensus Outcomes. *Mindfulness (N Y)* (2023) 14:279–292. doi: 10.1007/s12671-022-02015-1
41. Kielty ML, Gilligan TD, Staton AR. Whole-School Approaches to Incorporating Mindfulness-Based Interventions: Supporting the Capacity for Optimal Functioning in School Settings. *Child Educ* (2017) 93:128–135. doi: 10.1080/00094056.2017.1300491
42. Sheinman N, Hadar LL, Gafni D, Milman M. Preliminary Investigation of Whole-School Mindfulness in Education Programs and Children's Mindfulness-Based Coping Strategies. *J Child Fam Stud* (2018) 27:3316–3328. doi: 10.1007/s10826-018-1156-7
43. Steinebach C, Langer ÁI, Thuy TTM. "Enhancing Resilience in Youth: Sustainable Systemic Effects in Different Environments.," In: Steinebach C, Langer ÁI, editors. *Enhancing Resilience in Youth: Mindfulness-Based Interventions in Positive Environments*. Cham: Springer (2019). p. 3–17 doi: 10.1007/978-3-030-25513-8_1
44. Monsillion J, Zebdi R, Romo-desprez L. School Mindfulness-Based Interventions for Youth, and Considerations for Anxiety, Depression, and a Positive School Climate—A Systematic Literature Review. *Children* (2023) 10:861. https://www.mdpi.com/2227-9067/10/5/861
45. Gates GS, Gilbert B. "Mindful School Leadership: Guidance from Eastern Philosophy on Organizing Schools for Student Success.," In: Schonert-Reichl K, Roeser RW, editors. *Handbook of Mindfulness in Education*. Cham: Springer (2016). p. 251–267 doi: 10.1007/978-1-4939-3506-2_16
46. Irarrázaval M. "Recommendations for Mindfulness Interventions in the Educational Context.," In: Steinebach C, Langer ÁI, editors. *Enhancing Resilience in Youth: Minfulness-Based Interventions in Positive Environments*. Cham: Springer (2019) https://doi.org/10.1007/978
47. Tarrasch R. Mindful Schooling: Better Attention Regulation among Elementary School Children who Practice Mindfulness as Part of their School Policy. *J Cogn Enhanc* (2017) 1:84–95. doi: 10.1007/s41465-017-0024-5
48. Kuyken W, Ball S, Crane C, Ganguli P, Jones B, Montero-Marin J, Nuthall E, Raja A, Taylor L, Tudor K, et al. Effectiveness of universal school-based mindfulness training compared with normal school provision on teacher mental health and school climate: Results of the MYRIAD cluster randomised controlled trial. *Evid Based Ment Health* (2022) 25:125–134. doi: 10.1136/ebmental-2022-300424
49. Ergas O. Education and Mindfulness Practice: Exploring a Dialog Between Two

Traditions. *Mindfulness (N Y)* (2019) 10:1489–1501. doi: 10.1007/s12671-019-01130-w
50. Bhaskar R, Danermark B, Price L. *Interdisciplinarity and Wellbeing: A Critical Realist General Theory of Interdisciplinarity*. London and New York, NY: Routledge (2018). https://www.amazon.co.uk/Interdisciplinarity-Wellbeing-Critical-Realist-Routledge/dp/0415403715/ref=sr_1_1?crid=4O3D8WANEIEM&keywords=critical+realism+wellbeing&qid=1662303252&s=books&sprefix=critical+realism+wellbeing+%2Cstripbooks%2C70&sr=1-1
51. Pilgrim D. Mental health, subjectivities and forms of neuroscience: a critical realist examination. *Soc Theory Heal* (2019) 17:140–157. https://doi.org/10.1057/s41285-019-00088-y%0AORIGINAL ARTICLE%0AMental
52. Næss P. 'Demi-regs', probabilism and partly closed systems. *J Crit Realis* (2019) 18:475–486. doi: 10.1080/14767430.2019.1644951
53. Lawson T. *Economics and Reality*. London: Routledge (1997).
54. Danermark B, Ekström M, Karlsson JC. *Explaining Society: Critical Realism in the Social Sciences*. London and New York, NY: Routledge (2019).
55. Collier A. *Critical Realism: An Introduction to Roy Bhaskar's Philosophy*. London: Verso (1994).
56. Elder-Vass D. *The Causal Power of Social Structures: Emergence, Structure and Agency*. Cambridge: Cambridge University Press (2010). https://www.cambridge.org/core/books/causal-power-of-social-structures/DC90FA9DAB2FDCFFF9E3D8E98A7D9585
57. Sayer A. *Realism and Social Science*. London: SAGE (2000).
58. Sawyer S, Raniti M. How School Systems Can Improve Health and Well-Being. Topic Brief: Mental Health. (2023) https://www.who.int/health-topics/mental-health#tab=tab_2
59. Tikly L. What works, for whom, and in what circumstances? Towards a critical realist understanding of learning in international and comparative education. *Int J Educ Dev* (2015) 40:237–249. doi: 10.1016/j.ijedudev.2014.11.008
60. Weare K. Where Have We Been and Where Are We Going with Mindfulness in Schools? *Mindfulness (N Y)* (2023)293–299. doi: 10.1007/s12671-023-02086-8
61. Mischenko PP, Nicholas-Hoff P, Schussler DL, Iwu J, Jennings PA. Implementation barriers and facilitators of a mindfulness-based social emotional learning program and the role of relational trust: A qualitative study. *Psychol Sch* (2022) 59:1643–1671. doi: 10.1002/pits.22724
62. Langford R, Bonell C, Komro K, Murphy S, Magnus D, Waters E, Gibbs L, Campbell R. The Health Promoting Schools Framework: Known Unknowns and an Agenda for Future Research. *Heal Educ Behav* (2017) 44:463–475. doi: 10.1177/1090198116673800
63. Engel GL. The need for a new medical model: A challenge for biomedicine. *Science (80-)* (1977) 196:129–136. https://www.science.org/doi/abs/10.1126/science.847460
64. Pilgrim D. Some implications of critical realism for mental health research. *Soc Theory Heal* (2014) 12:1–21. doi: 10.1057/sth.2013.17
65. Danermark B. Applied interdisciplinary research: a critical realist perspective. *J Crit Realis* (2019) 18:368–382. doi: 10.1080/14767430.2019.1644983
66. Rupprecht S, Koole W, Chaskalson M, Tamdjidi C, West M. Running too far ahead? Towards a broader understanding of mindfulness in organisations. *Curr Opin Psychol* (2019) 28:32–36. doi: 10.1016/j.copsyc.2018.10.007
67. Donald JN, Bradshaw EL, Ryan RM, Basarkod G, Ciarrochi J, Duineveld JJ, Guo J, Sahdra BK. Mindfulness and Its Association With Varied Types of Motivation: A

Systematic Review and Meta-Analysis Using Self-Determination Theory. *Personal Soc Psychol Bull* (2020) 46:1121–1138. doi: 10.1177/0146167219896136
68. Klingbeil DA, Renshaw TL, Willenbrink JB, Copek RA, Chan KT, Haddock A, Yassine J, Clifton J. Mindfulness-based interventions with youth: A comprehensive meta-analysis of group-design studies. *J Sch Psychol* (2017) 63:77–103. doi: 10.1016/j.jsp.2017.03.006
69. Maynard BR, Solis MR, Miller VL, Brendel KE. Mindfulness-based interventions for improving cognition, academic achievement, behavior, and socioemotional functioning of primary and secondary school students. *Campbell Syst Rev* (2017) 13:1–144. doi: 10.4073/csr.2017.5
70. Shute RH. School-Based Mindfulness Interventions. *Oxford Res Encycl Educ* (2019) https://doi.org/10.1093/acrefore/9780190264093.013.979 [Accessed June 8, 2023]
71. Baelen RN, Gould LF, Felver JC, Schussler DL, Greenberg MT. Implementation Reporting Recommendations for School-Based Mindfulness Programs. *Mindfulness (N Y)* (2022)255–278. doi: 10.1007/s12671-022-01997-2
72. Lavy S, Berkovich-Ohana A. From Teachers' Mindfulness to Students' Thriving: the Mindful Self in School Relationships (MSSR) Model. *Mindfulness (N Y)* (2020) 11:2258–2273. doi: 10.1007/s12671-020-01418-2
73. Coulombe S, Hardy K, Goldfarb R. Promoting wellbeing through positive education: A critical review and proposed social ecological approach. *Theory Res Educ* (2020) 18:295–321. doi: 10.1177/1477878520988432
74. Siegel RD. Positive Psychology: Harnessing the power of happiness, mindfulness, and inner strength. (2013) https://www.health.harvard.edu/mind-and-mood/positive-psychology-harnessing-the-power-of-happiness-mindfulness-and-inner-strength#:~:text=Add To Cart-,Positive Psychology%3A Harnessing the power of happiness%2C mindfulness%2C and,being in numerous scienti
75. Feuerborn LL, Gueldner B. Mindfulness and Social-Emotional Competencies: Proposing Connections Through a Review of the Research. *Mindfulness (N Y)* (2019) 10:1707–1720. doi: 10.1007/s12671-019-01101-1
76. Kazanjian CJ. Mindfulness diligence: Supporting the culturally relative self-actualization processes of diverse groups of youth. *Humanist Psychol* (2020) 50:234–255. doi: 10.1037/hum0000192
77. Shute RH. Schools, mindfulness, and metacognition: A view from developmental psychology. *Int J Sch Educ Psychol* (2019) 7:123–136. doi: 10.1080/21683603.2018.1435322
78. Griffith MT. Exploring Mindfulness as a Social and Emotional Learning [SEL] Intervention: An Action Research Case Study. University of South Carolina (2022). https://scholarcommons.sc.edu/etd/7072
79. Dong X, Geng L. The role of mindfulness and meaning in life in adolescents' dispositional awe and life satisfaction: the broaden-and-build theory perspective. *Curr Psychol* (2022) doi: 10.1007/s12144-022-03924-z
80. Garland EL, Farb NA, R. Goldin P, Fredrickson BL. Mindfulness Broadens Awareness and Builds Eudaimonic Meaning: A Process Model of Mindful Positive Emotion Regulation. *Psychol Inq* (2015) 26:293–314. doi: 10.1080/1047840X.2015.1064294
81. Garland E, Gaylord S, Park J. The Role of Mindfulness in Positive Reappraisal. *Explor J Sci Heal* (2009) 5:37–44. doi: 10.1016/j.explore.2008.10.001
82. Madonna J. Mindfulness Practitioners Clarify the Concept of "Re-Perceiving": A Qualitative Interview Study. (2018) 3:111–133.
83. Niksirat KS, Silpasuwanchai C, Ahmed MMH, Cheng P, Ren X. A framework for interactive mindfulness meditation using attention-regulation process. *Conf Hum*

Factors Comput Syst - Proc (2017) 2017-May:2672–2684. doi: 10.1145/3025453.3025914
84. Sipe WEB, Eisendrath SJ. Mindfulness-based cognitive therapy: Theory and practice. *Can J Psychiatry* (2012) 57:63–69. doi: 10.1177/070674371205700202
85. Garrison KA, Zeffiro TA, Scheinost D, Constable RT, Brewer JA. Meditation leads to reduced default mode network activity beyond an active task. *Cogn Affect Behav Neurosci* (2015) 15:712–720. doi: 10.3758/s13415-015-0358-3
86. O'Campo P, Kirst M, Tsamis C, Chambers C, Ahmad F. Implementing successful intimate partner violence screening programs in health care settings: Evidence generated from a realist-informed systematic review. *Soc Sci Med* (2011) 72:855–866. doi: 10.1016/j.socscimed.2010.12.019
87. Lindsay EK, Creswell JD. Mechanisms of mindfulness training: Monitor and Acceptance Theory (MAT). *Clin Psychol Rev* (2017) 51:48–59. doi: 10.1016/j.cpr.2016.10.011
88. Guendelman S, Medeiros S, Rampes H. Mindfulness and emotion regulation: Insights from neurobiological, psychological, and clinical studies. *Front Psychol* (2017) 8: doi: 10.3389/fpsyg.2017.00220
89. Campbell M. A Case Study on Youth Perspectives Surrounding Mindfulness Practices. California State University, San Marcos (2023). https://scholarworks.calstate.edu/downloads/2514nt57f
90. Schultz PP, Ryan RM. "The 'Why,' 'What,' and 'How' of Healthy Self-Regulation: Mindfulness and Well-Being from a Self-Determination Theory Perspective,." In: Ostafin BD, Robinson MD, Meier BP, editors. *Handbook of Mindfulness and Self-Regulation*. Cham: Springer (2015). p. 1–301 doi: 10.1007/978-1-4939-2263-5
91. Ryan RM, Donald JN, Bradshaw EL. Mindfulness and Motivation: A Process View Using Self-Determination Theory. *Curr Dir Psychol Sci* (2021) 30:300–306. doi: 10.1177/09637214211009511
92. Reibel D, McCown D. "Mindfulness-Based Stress Reduction. Theory, practice and evidence base.," In: Ivtzan I, editor. *Handbook of Mindfulness-Based Programmes Mindfulness Interventions from Education to Health and Therapy*. London: Routledge (2019) https://www.taylorfrancis.com/chapters/edit/10.4324/9781315265438-4/mindfulness-based-stress-reduction-diane-reibel-donald-mccown
93. Micklitz K, Wong G, Howick J. Mindfulness-based programmes to reduce stress and enhance well-being at work: A realist review. *BMJ Open* (2021) 11:1–16. doi: 10.1136/bmjopen-2020-043525
94. Roeser RW, Taylor C, Harrison J. "Self-enhancement through self-transcendence: Towards mindful middle schools for learning and teaching.," In: Roney K, Lipka RP, editors. *Middle School Curriculum: Voices of the Self-Enhancing School*. Charlotte, NC: Information Age Publishing (2013) https://www.infoagepub.com/products/Middle-Grades-Curriculum
95. García-Rubio C, Herrero M, Luna-Jarillo T, Albert J, Rodríguez-Carvajal R. Effectiveness and mechanisms of change of a mindfulness-based intervention on elementary school children: A cluster-randomized control trial. *J Sch Psychol* (2023) 99:101211. doi: 10.1016/j.jsp.2023.04.001
96. Mendelson T, Dariotis JK, Gould LF, Smith ASR, Smith AA, Gonzalez AA, Greenberg MT. Implementing mindfulness and yoga in urban schools: A community-academic partnership. *J Child Serv* (2013) 8:276–291. doi: 10.1108/JCS-07-2013-0024
97. Roeser RW, Mashburn AJ, Skinner EA, Choles JR, Taylor C, Rickert NP, Pinela C, Robbeloth J, Saxton E, Weiss E, et al. Mindfulness Training Improves Middle School Teachers' Occupational Health, Well-Being, and Interactions With Students in Their

Most Stressful Classrooms. *J Educ Psychol* (2022) 114:408–425. doi: 10.1037/edu0000675
98. Young T. “Additional Mechanisms of wellbeing. How does mindfulness increase wellbeing.,” In: Ivtzan I, Lomas T, editors. *Mindfulness in Positive Psychology: The Science of Meditation and Wellbeing*. London and New York: Routledge (2016) https://www.routledge.com/Mindfulness-in-Positive-Psychology-The-Science-of-Meditation-and-Wellbeing/Ivtzan-Lomas/p/book/9781138808515#:~:text=Description,research and application of mindfulness.
99. Pilgrim D. *Critical Realism for Psychologists*. Abingdon and New York, NY: Routledge (2020).
100. Braun SS, Roeser RW, Mashburn AJ, Skinner E. Middle School Teachers’ Mindfulness, Occupational Health and Well-Being, and the Quality of Teacher-Student Interactions. *Mindfulness (N Y)* (2019) 10:245–255. doi: 10.1007/s12671-018-0968-2
101. Voight A, Nation M. Practices for Improving Secondary School Climate: A Systematic Review of the Research Literature. *Am J Community Psychol* (2016) 58:174–191. doi: 10.1002/ajcp.12074
102. Bonell C, Fletcher A, Jamal F, Wells H, Harden A, Murphy S, Thomas J. Theories of how the school environment impacts on student health: Systematic review and synthesis. *Heal Place* (2013) 24:242–249. doi: 10.1016/j.healthplace.2013.09.014
103. Albrecht N, Veall A. Wellness: A Conceptual Framework for School-based Mindfulness Programs. *Int J Heal Wellness Soc* (2016) 4: doi: 10.13140/RG.2.2.28103.09120
104. Langer ÁI, Steinebach C, García-Rubio C, Andreu CI, Torres-Díaz L. “Looking for a broad framework for the integration of mindfulness-based interventions in the educational system.,” In: Steinebach C, Langer ÁI, editors. *Enhancing Resilience in Youth: Mindfulness-Based Interventions in Positive Environments*. Cham: Springer International Publishing (2019) doi: 10.1007/978-3-030-25513-8
105. Hutchinson JK, Huws JC, Dorjee D. *Exploring experiences of children in applying a school-based mindfulness programme to their lives*. (2018). 3935–3951 p. doi: 10.1007/s10826-018-1221-2
106. Wilde S, Sonley A, Crane C, Ford T, Raja A, Robson J, Taylor L, Kuyken1 W. Mindfulness Training in UK Secondary Schools: a Multiple Case Study Approach to Identification of Cornerstones of Implementation. *Mindfulness (N Y)* (2019) 10:376–389. https://doi.org/10.1007/s12671-018-0982-4
107. Hudson KG, Lawton R, Hugh-Jones S. Factors affecting the implementation of a whole school mindfulness program: A qualitative study using the consolidated framework for implementation research. *BMC Health Serv Res* (2020) 20:1–13. doi: 10.1186/s12913-020-4942-z
108. Cheek J, Abrams EM, Lipschitz DL, Vago DR, Nakamura Y. Creating Novel School-Based Education Programs to Cultivate Mindfulness in Youth: What The Letters Told Us. *J Child Fam Stud* (2017) 26:2564–2578. doi: 10.1007/s10826-017-0761-1
109. Thomas G, Atkinson C. Perspectives on a whole class mindfulness programme. *Educ Psychol Pract* (2017) 33:231–248. doi: 10.1080/02667363.2017.1292396
110. Norton KR, Griffith GM. The Impact of Delivering Mindfulness-Based Programmes in Schools: A Qualitative Study. *J Child Fam Stud* (2020) 29:2623–2636. doi: 10.1007/s10826-020-01717-1
111. Coholic D, Schwabe N, Lander K. A Scoping Review of Arts-Based Mindfulness Interventions for Children and Youth. *Child Adolesc Soc Work J* (2020) 37:511–526.

doi: 10.1007/s10560-020-00657-5
112. Huppert FA, Johnson DM. A controlled trial of mindfulness training in schools: The importance of practice for an impact on well-being. *J Posit Psychol* (2010) 5:264–274. doi: 10.1080/17439761003794148
113. Lyons KE, DeLange J. "Mindfulness Matters in the Classroom: The Effects of Mindfulness Training on Brain Development and Behavior in Children and Adolescents.," In: Schonert-Reichl K, Roeser RW, editors. *Handbook of Mindfulness in Education, Mindfulness*. Cham (2016). p. 271–283 doi: 10.1007/978-1-4939-3506-2_17
114. Maloney JE, Lawlor MS, Schonert-Reichl KA, Whitehead J. "A Mindfulness-Based Social and Emotional Learning Curriculum for School-Aged Children: The MindUP Program.," In: Schonert-Reichl K, Roeser RW, editors. *Handbook of Mindfulness in Education Integrating Theory and Research into Practice*. Cham: Springer (2016). p. 313–334 doi: 10.1007/978-1-4939-3506-2_20
115. Lantieri L, Nambiar M, Harnett S, Kyse EN. "Cultivating Inner Resilience in Educators and Students: The Inner Resilience Program.," In: Schonert-Reichl K, Roeser RW, editors. *Handbook of Mindfulness in Education, Mindfulness in Behavioral Health*. Cham: Springer (2016). p. 119–132 doi: 10.1007/978-1-4939-3506-2_8
116. Dariotis JK, Mirabal-Beltran R, Cluxton-Keller F, Gould LF, Greenberg MT, Mendelson T. A Qualitative Evaluation of Student Learning and Skills Use in a School-Based Mindfulness and Yoga Program. *Mindfulness (N Y)* (2016) 7:76–89. https://link.springer.com/article/10.1007/s12671-015-0463-y
117. Parto M, Besharat MA. Mindfulness, psychological well-being and psychological distress in adolescents: Assessing the mediating variables and mechanisms of autonomy and self-regulation. *Procedia - Soc Behav Sci* (2011) 30:578–582. doi: 10.1016/j.sbspro.2011.10.112
118. Van der Gucht K, Takano K, Raes F, Kuppens P. Processes of change in a school-based mindfulness programme: cognitive reactivity and self-coldness as mediators. *Cogn Emot* (2018) 32:658–665. doi: 10.1080/02699931.2017.1310716
119. Zhang Y, Razza R, Wang Q, Bergen-Cico D, Liu Q. Mechanisms of Change Underlying Mindfulness-Based Practice Among Adolescents. *Mindfulness (N Y)* (2022) 13:1445–1457. doi: 10.1007/s12671-022-01881-z
120. Siegel DJ, Siegel MW, Parker SC. "Internal Education and the Roots of Resilience: Relationships and Reflection as the New R's of Education,." *Handbook of Mindfulness in Education Integrating Theory and Research into Practice*. Cham: Springer (2016). p. 47–63 doi: 10.1007/978-1-4939-3506-2_4
121. Lombas AS, Jiménez TI, Arguís-Rey R, Hernández-Paniello S, Valdivia-Salas S, Martín-Albo J. Impact of the Happy Classrooms Programme on Psychological Well-being, School Aggression, and Classroom Climate. *Mindfulness (N Y)* (2019) 10:1642–1660. doi: 10.1007/s12671-019-01132-8
122. Meyer L, Eklund K. The Impact of a Mindfulness Intervention on Elementary Classroom Climate and Student and Teacher Mindfulness: a Pilot Study. *Mindfulness (N Y)* (2020) 11:991–1005. doi: 10.1007/s12671-020-01317-6
123. Fung J, Kim JJ, Jin J, Chen G, Bear L, Lau AS. A Randomized Trial Evaluating School-Based Mindfulness Intervention for Ethnic Minority Youth: Exploring Mediators and Moderators of Intervention Effects. *J Abnorm Child Psychol* (2019) 47:1–19. doi: 10.1007/s10802-018-0425-7
124. Perry-Parrish C, Copeland-Linder N, Webb L, Sibinga EM. Mindfulness-based approaches for children and youth. *Curr Probl Pediatr Adolesc Health Care* (2016)

46:172–178. doi: 10.1016/j.cppeds.2015.12.006
125. Sibinga EMS, Perry-Parrish C, Chung S en, Johnson SB, Smith M, Ellen JM. School-based mindfulness instruction for urban male youth: A small randomized controlled trial. *Prev Med (Baltim)* (2013) 57:799–801. doi: 10.1016/j.ypmed.2013.08.027
126. Andreu CI, Araya-Véliz C, García-Rubio C. Benefits of a Mindfulness-based Intervention at School from the Perspective of At-risk Children. *Mindfulness (N Y)* (2021) 12:1611–1623. doi: 10.1007/s12671-021-01624-6
127. Schussler DL, Oh Y, Mahfouz J, Levitan J, Frank JL, Broderick PC, Mitra JL, Berrena E, Kohler K, Greenberg MT. Stress and Well-Being: A Systematic Case Study of Adolescents' Experiences in a Mindfulness-Based Program. *J Child Fam Stud* (2021) 30:431–446. doi: 10.1007/s10826-020-01864-5
128. McKeering P, Hwang YS. A Systematic Review of Mindfulness-Based School Interventions with Early Adolescents. *Mindfulness (N Y)* (2019) 10:593–610. doi: 10.1007/s12671-018-0998-9
129. Hwang YS, Bartlett B, Greben M, Hand K. A systematic review of mindfulness interventions for in-service teachers: A tool to enhance teacher wellbeing and performance. *Teach Teach Educ* (2017) 64:26–42. doi: 10.1016/j.tate.2017.01.015
130. Phan ML, Renshaw TL, Caramanico J, Greeson JM, MacKenzie E, Atkinson-Diaz Z, Doppelt N, Tai H, Mandell DS, Nuske HJ. Mindfulness-Based School Interventions: a Systematic Review of Outcome Evidence Quality by Study Design. *Mindfulness (N Y)* (2022) 13:1591–1613. doi: 10.1007/s12671-022-01885-9
131. Lavelle Heineberg BD. "Promoting Caring: Mindfulness- and Compassion-Based Contemplative Training for Educators and Students.," In: Schonert-Reichl K, Roeser RW, editors. *Handbook of Mindfulness in Education*. Cham: Springer (2016). p. 285–294 doi: 10.1007/978-1-4939-3506-2_18
132. World Bank. World Development Indicators. Washington DC: World Bank (2023). https://databank.worldbank.org/source/world-development-indicators
