# Supplementary material for: A protocol for a critical realist synthesis of school mindfulness interventions designed to promote pupils’ mental wellbeing
Source: Front Public Health. 2024 Jan 9;11:1309649. doi: 10.3389/fpubh.2023.1309649 (PMC10803664; doi:10.3389/fpubh.2023.1309649)
Supplement: Supplementary file 6 [file Data_Sheet_6.PDF]

Supplementary Material 6: Research Evidence Extraction/appraisal Tool for Papers included in the Critical Realist Analysis Phase

**Analyse Phase**						
	Article Title:					
Authors						
Journal/ Edited Collection, PhD						
Setting						
Sample Size						
Objective of study clearly stated						
Study methodology or methodologies used						
Inclusion of sufficient data to assess validity of conclusions						
Data source						
Size of achieved sample and the population from which the sample is drawn						
Methods of measurement (data collection)						
Effect on behaviour						
Study type	☐ Meta-analysis	☐ Quasi-experimental	☐ Non-experimental	☐ Qualitative	☐ Meta-synthesis	☐ Realist /Critical Realist synthesis
Does this study apply to the population targeted the review question?				☐ Yes	☐ No	☐

**Strength of the study design**			
Is the sample size adequate and appropriate?	☐ Yes	☐ No	☐ NR
Are the study participants randomised?	☐ Yes	☐ No	☐ NR
Is there an intervention?	☐ Yes	☐ No	☐ NR
Is there a control group?	☐ Yes	☐ No	☐ NR
If there is more than one group, are the groups equally treated except for the intervention?	☐ Yes	☐ No	☐ NR
Is there an adequate description of the data collection methods?	☐ Yes	☐ No	☐ NR
**Study Results**			
Are the results clearly presented?	☐ Yes	☐ No	☐ NR
Is the interpretation/analysis provided	☐ Yes	☐ No	☐ NR
**Study Conclusions**			
Are the conclusions based on clearly presented results?	☐ Yes	☐ No	☐ NR
Are the study limitations identified and discussed?	☐ Yes	☐ No	☐

	Pertinent study findings and recommendations

Will the results answer the review questions?	☐ Yes	☐ No

**Evidence Rating**					
Strength of Evidence	☐ Level I	☐Level II	☐Level III	☐Level IV	☐Level V
Quality of Evidence (Check one)			☐High (A)	☐Good	☐Low (c)

Extraction form to guide data extraction of studies in a systematic review of the health and social impacts

Egan, M., Petticrew, M., Hamilton, V., and Ogilvie, D. Health impacts of new roads: A systematic review. American Journal of Public Health 2003, 93(9): 1463–71
